# Construction and validation of a multi-epitope in silico vaccine model for lymphatic filariasis by targeting *Brugia malayi*: a reverse vaccinology approach

**DOI:** 10.1186/s42269-023-01013-0

**Published:** 2023-03-24

**Authors:** Premnath Madanagopal, Sathya Muthusamy, Satya Narayan Pradhan, Prabhu Rajaiah Prince

**Affiliations:** 1 Department of Biotechnology, Anna University, Chennai, India; 2 The Hamburg Centre for Ultrafast Imaging (CUI), University of Hamburg, Hamburg, Germany; 3 Institute for Biochemistry and Molecular Biology, Laboratory for Structural Biology of Infection and Infammation, University of Hamburg, c/o DESY, 22603, Hamburg, Germany

**Keywords:** Immunoinformatics, Lymphatic filariasis, *Brugia malayi*, Molecular docking, Multi-epitope peptide-based vaccine, Reverse vaccinology

## Abstract

**Background:**

Lymphatic filariasis (LF), often referred to as elephantiasis, has been identified as one of the 17 neglected tropical diseases by the World Health Organization. Currently, there are no vaccines available to treat this infection in humans. Therefore, with the objective of devising a novel preventive measure, we exploited an immunoinformatics approach to design a multi-epitope-based subunit vaccine for LF, that can elicit a variety of immune responses within the host. In this study, different B cell, T_C_ cell, and T_H_ cell-binding epitopes were screened from the antigenic proteins of *Brugia malayi* and they were passed through several immunological filters to determine the optimal epitopes.

**Results:**

As a result, 15 CD8+, 3 CD4+, and 3 B cell epitopes were found to be prominent, antigenic, non-toxic, immunogenic and non-allergenic. The presence of conformational B cell epitopes and cytokine-inducing epitopes confirmed the humoral and cell-mediated immune response that would be triggered by the constructed vaccine model. Following that, the selected epitopes and TLR-4-specific adjuvant were ligated by appropriate peptide linkers to finalize the vaccine construct. Protein–protein docking of the vaccine structure with the TLR4 receptor predicted strong binding affinity and hence putatively confirms its ability to elicit an immune response. Further, the efficiency of the vaccine candidate to provide a long-lasting protective immunity was assessed by in silico immune simulation. The reverse translated vaccine sequence was also virtually cloned in the pET28a (+) plasmid after the optimization of the gene sequence.

**Conclusion:**

So taken together, by monitoring the overall in silico assessment, we hypothesize that our engineered peptide vaccine could be a viable prophylactic approach in the development of vaccines against the threat of human lymphatic filariasis.

**Graphical Abstract:**

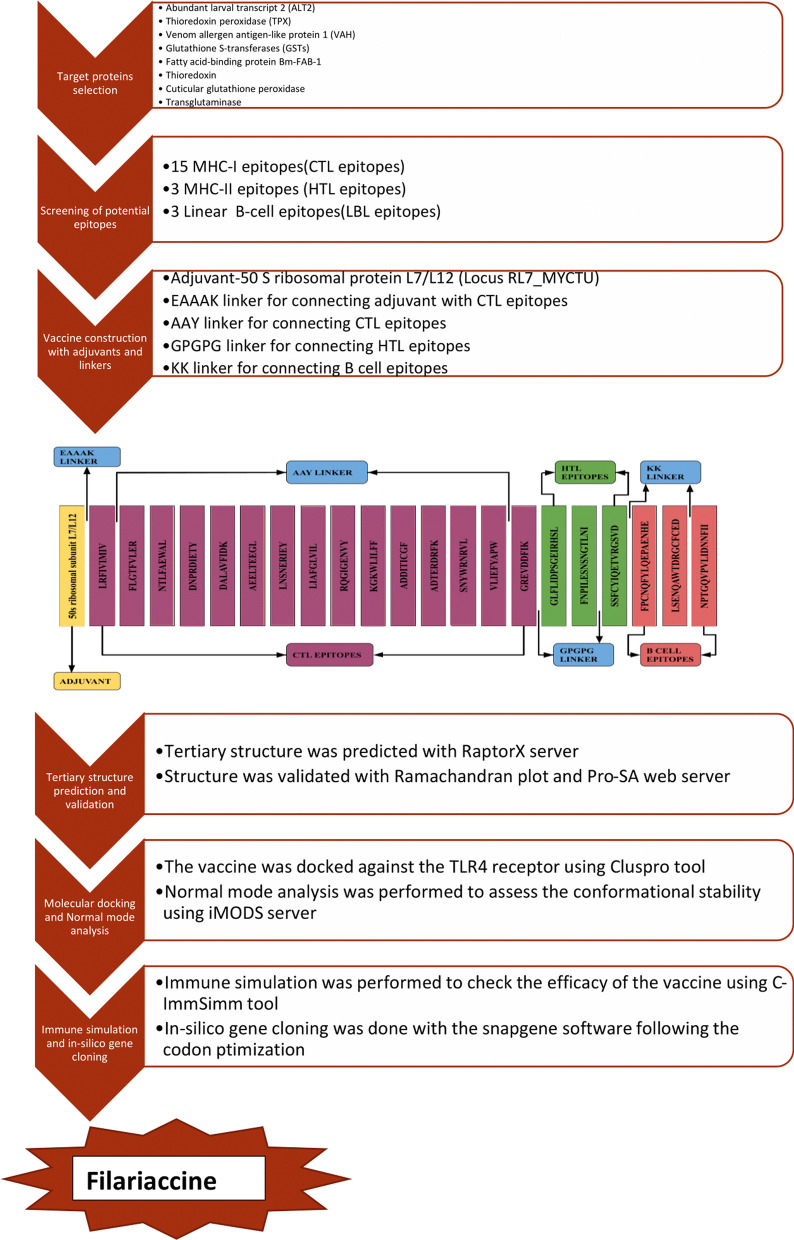

**Supplementary Information:**

The online version contains supplementary material available at 10.1186/s42269-023-01013-0.

## Highlights

- A novel computational approach was adopted for the development of a vaccine model against lymphatic filariasis.
- The potential epitopes were screened to test their antigenicity, toxicity, immunogenicity, and allergenicity.
- The prophylactic vaccine model was constructed with appropriate peptide linkers and the adjuvant—50 s ribosomal protein L7/L12.
- The tertiary structure of the vaccine model was validated with Ramachandran plot, ProSA, etc.
- Molecular docking against TLR4 receptor and immune simulation studies were performed to test the efficacy of the vaccine.

**Supplementary Information:**

The online version contains supplementary material available at 10.1186/s42269-023-01013-0.

## Background

Human lymphatic filariasis (LF) is a neglected tropical disease (NTD) caused by either of the three filarial nematodes such as *Wuchereria bancrofti*, *Brugia malayi,* and *Brugia timori* (Gorai et al. 2022; Specht et al. 2019). The concept of neglected tropical diseases (NTDs) emerged more than a decade ago and was recognized as a valid term to use for describing illnesses that are largely confined to the poor (Molyneux et al. 2017). Globally, the World Health Organization (WHO) has identified seventeen such neglected tropical diseases (Additional file 1: Table S1) that affect approximately 1 billion of the world’s poorest people and another 1 billion who are at the risk of getting an infection in the near future. Due to the lack of willingness to manufacture prophylactic vaccines for the benefit of such downtrodden communities, research and therapeutic interventions for these diseases are highly neglected, thereby becoming the cause as well as the result of poverty. Among the 17 neglected tropical diseases, lymphatic filariasis (LF) is endemic to about 80 tropical and subtropical countries, infecting approximately 120 million people (Wynd et al. 2007). According to WHO, about 2.8 million disability-adjusted life-years are attributed to lymphatic filariasis alone, thereby becoming the second most common cause of long-term disability.

Among the three filarial parasites, *Wuchereria bancrofti* is responsible for about 90% of the infections followed by *Brugia malayi*, and then *Brugia timori* (Ram 2016). The WHO has taken several measures to bring the disease under control, like the MDA (mass drug administration) programme in which whole communities are treated with anti-helminthic drugs at regular intervals and transmission assessment surveys (TAS) are undertaken to promote early diagnosis of the infection in acute stages (Riches et al. 2020). Apart from this, increasing efforts are also made to control the vector-borne transmission of the disease by mosquitoes (Deshpande et al. 2020). Though the MDA approach has significantly reduced the global death toll due to LF, this current strategy has certain concerns with regard to its long-term benefits and prevention of future infections. Hence, a more sustainable approach would be the development of a prophylactic vaccine against LF which would prevent the relapse or re-occurrence of the disease, besides inducing herd immunity within the population (Kalyanasundaram et al. 2020). Though several research groups have attempted to develop a vaccine using different filarial antigens (Babayan et al. 2012; Morris et al. 2013), there exists no commercially licensed vaccine for the cure of LF till now (Kalyanasundaram et al. 2020). And there are obvious reasons contributing to the difficulties in the construction of a potential vaccine against LF such as (i) lack of appropriate animal models to conduct research on LF, (ii) the complexity in the life-cycle of the parasite, (iii) the limited evidence to prove the existence of natural protective immunity in the host, (iv) the complicated immune responses in the host against the parasite, and (v) the paucity in information on the characteristics of a protective immune response in animals as well as humans (Morris et al. 2013).

The conventional strategy for developing a vaccine would be to use a live attenuated pathogen or a combination of protein complexes that are randomly selected from the parasite after extraction and purification, in order to stimulate an immune response in the host (Gorai et al. 2022). Such traditional methods used in vaccinology are labour-intensive, time-consuming, and also expensive due to the huge amount of money spent on the failed trials, let alone causing virulence reversion in some individuals (Gorai et al. 2022). However, these limitations are being outdone in the modern vaccines that are developed by reverse vaccinology technique and it has emerged as a promising field in immunology. In this reverse vaccinology method, instead of culturing the parasite, its genomic information including its epitope map is used for the construction of the vaccine (Gorai et al. 2022). This novel strategy of designing multi-epitope-based vaccines has been found successful for the development of vaccines against dengue virus (Ali et al. 2017; Fadaka et al. 2021), SARS-CoV-2 (Al Zamane et al. 2021; Ansori et al. 2021; Fahmi et al. 2021) and onchocerciasis (Shey et al. 2019) which is also a parasitic disease. Hence in our research, we intend to adopt a similar reverse vaccinology approach for the construction of an in silico vaccine model for the cure of lymphatic filariasis. Despite the fact that several bioinformatics tools and online servers are available for performing various screening tests and analyses, the selection of appropriate tools with relevance to our target organism, let alone its advantages, and limitations is crucial for the accuracy of results.

Though *W. bancrofti* is the major filarial parasite causing 90% of the infections, the comparison of selective well-characterized genes indicates that *W. bancrofti* and *B. malayi* share greater than 95% homology between them. Additionally, *B. malayi* is largely responsible for the infections in south-east Asian countries that hold one-third of the disease burden (Wynd et al. 2007). Moreover, the availability of the complete genome, transcriptome, proteome, and secretome data for the *Brugia malayi* parasite indicates that it is possible to develop an effective vaccine against LF as a prophylactic measure (Kalyanasundaram et al. 2020). Due to these reasons and by means of a curiosity-driven approach, *Brugia malayi* was selected as our target organism. In our research, we aim to develop a multi-epitope-based multivalent vaccine in order to prevent the LF parasite from exploiting multiple pathways to evade the host immune system. Hence, the following candidate antigens homologous in both the species—*B. malayi* and *W. Bancrofti*, were selected for screening the epitopes namely abundant larval transcript 2 (ALT2), thioredoxin peroxidase (TPX), venom allergen antigen-like protein 1(VAH), and glutathione S-transferases (GSTs). A few more antigens were chosen based on the criterion that the proteins get expressed on the surface of the infective stages of the parasite, thereby becoming readily accessible to the human immune system (Kalyanasundaram et al. 2020) Those antigens are: the embryonic fatty acid-binding protein Bm-FAB-1, thioredoxin (Gorai et al. 2022), cuticular glutathione peroxidase or cuticular glycoprotein gp29, and transglutaminase. To trigger early activation of the innate immunity and to induce broader adaptive protection, peptide antigens require an adjuvant. Some recent studies have shown promising results by using analogs of TLR-4 or tuftsin to improve vaccine protection in LF (Chauhan et al. 2017; Paul et al. 2018; Khatri et al. 2018), because TLR4 involvement has been linked to protective immunity (Shey et al. 2019; Kerepesi et al. 2005). Hence, the 50 S ribosomal protein L7/L12 (Locus RL7_MYCTU) was selected as an adjuvant (Accession no. P9WHE3) since it acts as an agonist for TLR4 (Gorai et al. 2022).

In this context, we present the in silico construction and validation studies for our multi-epitope-based vaccine-**“Filariaccine”**, which was designed with a number of HTL, CTL, and LBL epitopes, after carefully screening them for their antigenicity, immunogenicity, toxicity, and cross-reactivity.

## Methods

### Protein sequence retrieval for antigen prediction

As the first step in vaccine development, the genome of *Brugia malayi* was studied through literature, and to analyse the specific antigenic proteins, the complete amino acid sequences of the eight *Brugia malayi* proteins (glutathione S-transferase, embryonic fatty acid-binding protein, thioredoxin, abundant larval transcript-2, venom allergen antigen-like protein 1, peroxiredoxin 1, cuticular glycoprotein gp29, and transglutaminase) were extracted from NCBI (https://www.ncbi.nlm.nih.gov/protein) (Home-Protein n.d.) and UniProt (https://www.uniprot.org/) (Bateman 2019) databases in the FASTA format. Their respective accession numbers are glutathione S-transferase (UniProt: Q5GSK1), embryonic fatty acid-binding protein (NCBI: AAG09305), thioredoxin (NCBI: XP_042935624), abundant larval transcript-2 (NCBI: XP_001902122), venom allergen antigen-like protein 1 (NCBI: XP_042932260), peroxiredoxin 1 (UniProt: P48822), cuticular glycoprotein gp29 (NCBI: XP_001899550), and transglutaminase (NCBI: XP_001897232).

### Epitope prediction for vaccine construct

#### Prediction of cytotoxic T lymphocytes (CTL) epitope and MHC-I binding alleles

Predicting CTL epitopes was important for developing a vaccine as it plays an important role in cell-mediated immunity. The IEDB MHC-I processing tool (http://tools.iedb.org/processing/) (Fleri et al. 2017) was applied to identify 9-mer length of CTL epitopes with 49 frequently occurring MHC-I alleles (Additional file 2: Table S2) using the stabilized matrix method (SMM) keeping default values of 1 for the maximum precursor extension and 0.2 for the alpha factor. Prediction involves a combination of three approaches, proteasomal cleavage and processing, TAP transport efficiency, and MHC class I binding affinity in order to assess the outcome of antigen processing and presentation. The threshold was adjusted for MHC class I alleles at 50 nM (IC50 ≤ 50 strong binders) (Al Zamane et al. 2021).

#### Prediction of helper T-lymphocyte (HTL) epitope and MHC-II binding alleles

The induction of both humoral and cellular immune responses is aided by HTL responses. As a result, HTL epitopes are likely to play a key role in both prophylactic and immunotherapeutic vaccines. IEDB MHC-II Binding Predictions tool (http://tools.iedb.org/mhcii/) (Fleri et al. 2017) using the SMM method was used to predict 15-mer length of HTL epitopes against a set of 15 human HLAs (Additional file 3: Table S3). The prediction of HTL epitopes was based on affinity to MHC-II alleles, which can be deduced from the IC50 values and percentile ranks assigned to each predicted epitope. IC50 values for high-affinity peptides should be less than 50 nM. A value of 500 nM or less suggests intermediate affinity, while values of 5000 nM or more indicate poor affinity (Khatoon et al. 2017).

#### Linear B cell (LBL) epitopes prediction

To elicit a high expression of antigen-specific antibody production in the serum, B cell epitopes are important. Hence, the proteins were subjected to linear B cell epitope prediction using two different servers since the use of multiple tools in epitope prediction has been reported to improve the rate of true positives (Shey et al. 2019). Initially, epitopes for 16-mer were predicted using ABCPred, with a default threshold of 0.51 based on a recurrent neural network (http://www.imtech.res.in/raghava/abcpred/). Finally, FBCPred (El-Manzalawy et al. xxxx) (http://ailab-projects1.ist.psu.edu:8080/bcpred/predict.html) was utilized for the prediction of LBL epitopes with specificity adjusted to 75% which predicts epitopes of flexible length using the subsequence kernel.

### Antigenicity, allergenicity and toxicity prediction of the CTL, HTL, and LBL epitopes

The antigenicity of the proposed epitopes (CTL, HTL, and LBL) was verified using VaxiJen 2.0 server (http://www.ddg-pharmfac.net/vaxijen/) with a selected target organism as a parasite. This server is based on auto-cross-covariance (ACC) transformation of protein sequences into uniform vectors of principal amino acid properties. Peptides with an antigenicity threshold of ≥ 0.4 were considered to be potential immunogens (Al Zamane et al. 2021; Cuspoca et al. 2021). The allergenicity status of all CTL, HTL, and LBL epitopes was evaluated using the AllerTOP v.2.0 server (https://www.ddg-pharmfac.net/AllerTOP/) to decrease allergic responses to the selected epitopes. AllerTOP uses an auto-covariance transformation to normalize the alignment of peptides with immunogenic potential and includes an automatic and manual pull of cured allergenic and non-allergenic proteins, similar to VaxiJen 2.0 (Bateman 2019). Further, to rule out the toxic reactions caused by the interaction of peptides, we used ToxinPred (http://crdd.osdd.net/raghava/toxinpred/), an in silico approach based on machine learning technique and quantitative matrix using physicochemical properties of peptides.

### Immunogenicity prediction of the CTL epitopes

The immunogenicity of CTL epitopes was estimated using the MHC class I immunogenicity tool (http://tools.iedb.org/immunogenicity/) from the IEDB server. This tool was designed to identify the immunogenicity of peptides based on their amino acid position and other characteristics (Fleri et al. 2017).

### Prediction of cytokine-inducing HTL epitopes

The ability of the identified HTL epitopes to trigger various cytokines was investigated further (Nain et al. 2019). Interferon-gamma (IFN-γ), a cytokine that stimulates macrophages and natural killer cells and enhances the immune response to MHC antigens, is a key player in both adaptive and innate immune responses (Ali et al. 2017). Hence, T-helper epitopes were screened for IFN-γ epitopes using the IFNepitope server (https://webs.iiitd.edu.in/raghava/ifnepitope/). Also, HTL epitopes were screened to identify Interleukin-4 (IL4) stimulating epitopes using IL4pred (https://webs.iiitd.edu.in/raghava/il4pred/). IL4 had been shown to play a critical role in response to helminths and other extracellular parasites, and it is important for antibody class switching, haematopoiesis and inflammation, and the formation of proper effector T cell responses. Furthermore, HTL epitopes were scanned for interleukin-10 (IL-10) inducing peptides using IL-10Pred (https://webs.iiitd.edu.in/raghava/il10pred/) (Nagpal et al. 2017). IL-10 is an anti-inflammatory cytokine that keeps the immune response in balance, allowing infection to be expelled while causing the least amount of damage to the host.

### Population coverage

**Fig. 1:**
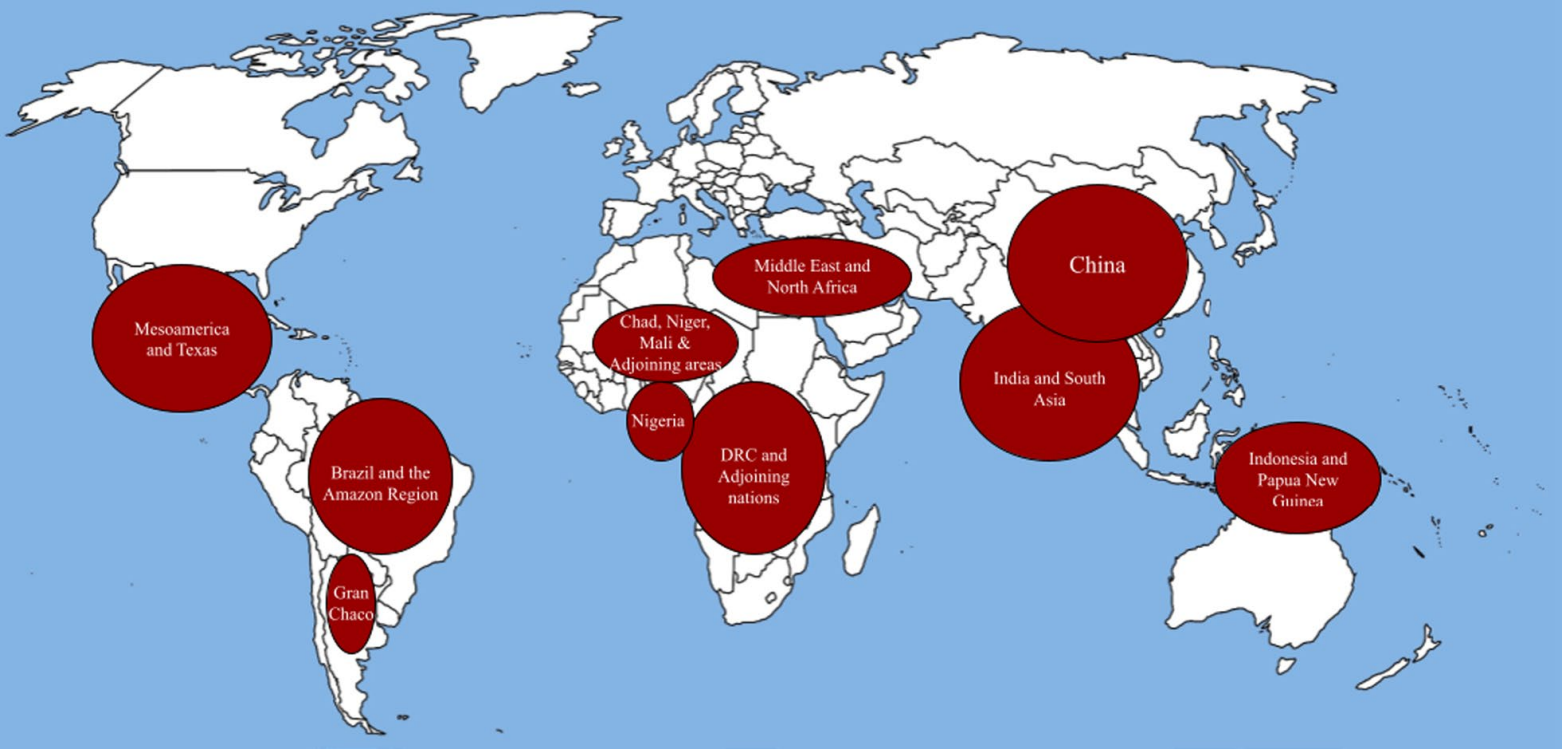
The 10 global hotspots for neglected tropical diseases (NTDs)

The shortlisted epitopes from the HLA class I, class II families, and their respective binding HLA alleles were subjected to the IEDB Population Coverage tool (http://tools.iedb.org/population/) (Fleri et al. 2017). Since the epitopes with different HLA alleles have distinct binding sites and it varies with different populations across the globe, the vaccines that are being designed should be able to cover a wide range of the world population. Therefore in this study, the population coverage of our CTL and HTL epitopes was estimated against the entire world and specific hotspots of NTD (Fig. 1) (Hotez 2014) to obtain and ensure a universal vaccine. The calculation option was set to “Class I and II combined” for the MHC alleles (Abdelmageed et al. 2020; Maleki et al. 2021).

### Construction of multi-epitope vaccine candidate sequence

All different HTL and CTL epitopes that were predicted by various immunoinformatic tools and immunological filters were used to design the multi-epitope vaccine with the help of linker proteins. Immunomodulatory molecules were introduced to vaccine constructs as safe vaccine adjuvants in order to amplify the immune system. Since peptides are not immunogenic on their own, they require an adjuvant to stimulate the immune system (Bin Sayed et al. 2020; Li et al. 2014). 50S ribosomal protein L7/L12 (accession no: P9WHE3.1) was chosen as an adjuvant to improve the immunogenicity of the vaccine since it is an agonist of TLR4 (Gorai et al. 2022; Das et al. 2021). Also, TLR4 agonists have been shown to induce strong T cell and antibody-mediated responses when used as adjuvants in vaccine candidates. The final vaccine was constructed by joining the adjuvants, MHC-I and MHC-II epitopes with EAAAK, GPGPG, and AAY, respectively, i.e. linker proteins.

### Evaluation of antigenicity, allergenicity, physicochemical properties, and solubility of the designed vaccine peptide

For the evaluation of antigenic features of constructed vaccine, VaxiJen v2.0 (http://www.ddg-pharmfac.net/vaxijen/) was used and the allergenicity of the vaccine was assessed from AllerTOP v.2.0 (https://www.ddg-pharmfac.net/AllerTOP/) (Dimitrov et al. 2014). The physiochemical properties of the vaccine were estimated using the ExPASy ProtParam (http://web.expasy.org/protparam/). These properties included amino acid composition, instability index, theoretical pI, aliphatic index, molecular weight, in vitro and in vivo half-life, and grand average of hydropathicity (GRAVY). The solubility of the vaccine construct was estimated using the ProteinSol (https://protein-sol.manchester.ac.uk/) (Hebditch et al. 2017).

### Cross-reactivity analysis with human proteomes

The BLASTP (https://blast.ncbi.nlm.nih.gov/Blast.cgi) search engine in the NCBI database was used to examine the sequential similarity of our epitope-based vaccine against human proteomes by applying the expected threshold value 10 and BLOSUM 62 matrix as a parameter.

### Secondary structure prediction

PSIPRED v4.0 (http://bioinf.cs.ucl.ac.uk/psipred/) (Buchan and Jones 2019) was used to predict the secondary structures of the designed vaccine candidate based on their primary sequences as input. This tool employs two feed-forward neural networks that execute an analysis of the output obtained from PSI-BLAST (Position-Specific Iterated-BLAST) (Ali et al. 2017).

### Tertiary structure construction, refinement, and validation

RaptorX (http://raptorx.uchicago.edu/ContactMap/) was used to infer the 3D model of our final vaccine. This server predicts tertiary structure using a deep learning technique that combines evolutionary coupling (EC) and sequence conservation information via an ultra-deep neural network constructed from two deep residual neural networks (Xu 2019). To improve the optimal 3D structure of the construct, the structure was refined by GalaxyRefine (https://galaxy.seoklab.org/cgi-bin/submit.cgi?type=REFINE) and the energy was then minimized using Swiss-PDB Viewer. To validate the modelled protein, the ProSA-web (Protein Structure Analysis) and PROCHECK (Ramachandran Plot Assessment) servers have been used. ProSA (https://prosa.services.came.sbg.ac.at/prosa.php) has been assigned with calculating an overall quality score for the given input structure. The PROCHECK server was used to obtain the Ramachandran plot, which determines the quality of the modelled structure.

### Conformational B cell epitope prediction

More than 90% of B cell epitopes are known to be discontinuous, meaning that they are made up of segments from the pathogen protein sequence that are brought together by the folding of the protein (Shey et al. 2019). Such epitopes are called conformational B cell epitopes. Hence, ElliPro (http://tools.iedb.org/ellipro/) was used to predict discontinuous (conformational) B cell epitopes for our validated 3D vaccine construct. This tool assigns a score to each output epitope based on the PI (Protrusion Index) value averaged over each epitope residue.

### Molecular docking and interaction studies with TLR4

Molecular docking is a crucial step to ensure the binding affinity of the vaccine with its receptor TLR4. For this purpose, the crystal structure of the TLR4 receptor (PDB code: 4G8A) was retrieved from the RCSB protein data bank (https://www.rcsb.org/) whose atomic resolution was 2.4 A°. The prepared tertiary structures of the vaccine and its receptor TLR4 were then subjected to a balanced docking method in the ClusPro 2.0 server (Desta et al. 2020; Vajda et al. 2017; Kozakov et al. 2017) (https://cluspro.bu.edu/) where rigid-body protein–protein docking was performed. Under the balanced docking method, the ClusPro server calculates the lowest weighted energies of the various docked complexes by employing the force field equation: *E* = 0.40Erep − 0.40Eatt + 600Eelec + 1.00EDARS (Kozakov et al. 2017). As a result, a number of docked clusters with their respective lowest energy weighted scores were obtained. The final selected cluster was visualized in the PyMoL molecular graphics system (Yuan et al. 2017), and the various non-bonded interactions occurring between the respective residues were deeply analysed using the Discovery studio 2021 client software (https://www.3ds.com/products-services/biovia/products/molecular-modeling-simulation/biovia-discovery-studio/).

### Normal mode analysis

Through iMODS (http://imods.chaconlab.org/), a normal mode analysis was performed to assess the conformational stability of the docked complex. It simulates protein collective motion by evaluating normal modes (NMA) in internal coordinates and predicting features including deformability, mobility profiles, eigenvalues, variance, and covariance map (Madanagopal et al. 2022).

### Immune simulation

To further validate the immunogenicity and immune response profile of the constructed vaccine, in silico immune simulation using the C-ImmSim server (https://kraken.iac.rm.cnr.it/C-IMMSIM/) (Yuan et al. 2017; Rapin et al. 2010; Castiglione et al. 2021) was performed. C-ImmSim is an agent-based model that predicts immunological interactions using a position-specific scoring matrix (PSSM) for immune epitope prediction and machine learning approaches. This server simulates the components of bone marrow, thymus, and lymph node (mammalian) by using the FASTA format of the vaccine. The vaccine’s ability to stimulate immune cells such as cytotoxic T cells, helper T cells, B cells, immunoglobulins, and cytokines was investigated. In the immune simulation, three dosages of injection were administrated at regular intervals of 4 weeks. Hence, the time step parameters were set as 1, 84, and 168 since one time step is equal to 8 h in real life. The total steps of the simulation were 1050 which approximates 1 year in real life. All the other parameters were kept as default.

### mRNA structure prediction

The secondary structure and the minimum free energy (ΔG) of folding and the positional entropy for the mRNA structure of the vaccine construct were predicted using the RNAfold web server (http://rna.tbi.univie.ac.at/cgi-bin/RNAWebSuite/RNAfold.cgi). A lower free energy value would indicate that the structure is thermodynamically stable.

### Codon optimization and in silico cloning

To express the multi-epitope vaccine design in a selected expression vector, the Java Codon Adaptation Tool (JCat) (https://www.jcat.de/) was used for reverse translation and codon optimization. For codon optimization, E. coli (strain K12) was used. The server gives a codon adaptation index (CAI), which indicates codon usage and GC content biases that can be used to evaluate protein expression levels. Finally, the cloning procedure was conducted by inserting the modified nucleotide sequence into the pET28a (+) expression vector using Snap Gene v4.2 software (https://snapgene.com/).

## Results

### Prediction of T cell epitopes and prioritization

In this study, CTL epitopes were predicted using the IEDB MHC-I processing tool, whereas HTL epitopes were predicted using the IEDB MHC-II Binding Predictions tool and both types of T cell epitopes were predicted by the SMM approach against a set of human reference HLAs with adjusted IC50 value less than 50 nM. A total of 15 CTL epitopes and 3 HTL epitopes were shortlisted for eight selected antigenic proteins by applying several immunological filters as shown in Tables 1 and 2. All of the optimal epitopes, as well as their antigenicity, positioning and binding alleles, are documented in Tables 3 and 4. In addition, for the best possible epitopes, the corresponding alleles which have a moderate binding affinity (IC50 < 500 nM) were also documented.

**Table 1:** Results of the finally shortlisted CTL epitopes

S. No	CTL epitope	Antigenicity	Allergenicity	Toxicity	Immunogenicity
1	LRFIVIMIV	ANTIGEN	NON-ALLERGEN	NON-TOXIN	0.26696
2	FLGTFVLER	ANTIGEN	NON-ALLERGEN	NON-TOXIN	0.25206
3	NTLFAEWAL	ANTIGEN	NON-ALLERGEN	NON-TOXIN	0.45635
4	DNPRDIETY	ANTIGEN	NON-ALLERGEN	NON-TOXIN	0.30254
5	DALAVFIDK	ANTIGEN	NON-ALLERGEN	NON-TOXIN	0.31145
6	AEELTEEGL	ANTIGEN	NON-ALLERGEN	NON-TOXIN	0.25769
7	LNSNERIEY	ANTIGEN	NON-ALLERGEN	NON-TOXIN	0.25683
8	LIAFGLVIL	ANTIGEN	NON-ALLERGEN	NON-TOXIN	0.26566
9	RQGIGENVY	ANTIGEN	NON-ALLERGEN	NON-TOXIN	0.29083
10	KGKWLILFF	ANTIGEN	NON-ALLERGEN	NON-TOXIN	0.32641
11	ADDITICGF	ANTIGEN	NON-ALLERGEN	NON-TOXIN	0.2785
12	ADTERDRFK	ANTIGEN	NON-ALLERGEN	NON-TOXIN	0.29671
13	SNYWRNRVL	ANTIGEN	NON-ALLERGEN	NON-TOXIN	0.3338
14	VLIEFYAPW	ANTIGEN	NON-ALLERGEN	NON-TOXIN	0.28101
15	GREVDDFIK	ANTIGEN	NON-ALLERGEN	NON-TOXIN	0.29308

**Table 2:** Results of the finally shortlisted cytokine-inducing HTL epitopes

S. No	HTL epitope	Antigen/non-antigen	Allergenicity	Toxicity	IFN server	IL10 server	IL4 server
1	GLFLIDPSGEIRHSL	ANTIGEN	NON-ALLERGEN	NON-TOXIN	POSITIVE	IL10 inducer	IL4 inducer
2	FNPILESNSNGTLNI	ANTIGEN	NON-ALLERGEN	NON-TOXIN	POSITIVE	IL10 inducer	IL4 inducer
3	SSFCYIQETVRGSVD	ANTIGEN	NON-ALLERGEN	NON-TOXIN	POSITIVE	IL10 inducer	IL4 inducer

**Table 3:** T_C_ epitopes with its binding allele (IC50 < 500 nM)

S.no	CTL epitope	Position	Allele	Proteasome score	TAP score	Processing score	IC50	Antigenicity (VaxiJen score)
1	LRFIVIMIV	Bm-FAB-1 (3–11)	HLA-C*12:03	0.92	0.29	1.21	10.9	1.6738
			HLA-C*07:01				32.6	
			HLA-C*06:02				232.2	
			HLA-C*14:02				235.3	
2	FLGTFVLER	Bm-FAB-1 (35–43)	HLA-C*12:03	1.06	0.58	1.64	32.4	0.8245
			HLA-C*03:03				78.1	
			HLA-C*14:02				215.1	
			HLA-A*68:01				218	
			HLA-A*31:01				313.7	
			HLA-A*02:01				328.2	
			HLA-B*15:02				437.8	
3	NTLFAEWAL	Bm-FAB-1 (94–102)	HLA-C*03:03	1.45	0.48	1.93	14.4	0.6420
			HLA-B*15:02				59.2	
			HLA-C*12:03				163	
			HLA-A*68:02				186.5	
			HLA-C*14:02				200.3	
			HLA-A*02:06				225.2	
4	DNPRDIETY	Bm-FAB-1 (137–145)	HLA-C*12:03	1.48	1.08	2.56	12.7	0.6283
			HLA-C*14:02				150.2	
			HLA-C*03:03				452.5	
			HLA-B*15:02				477.8	
5	DALAVFIDK	Thioredoxin family protein (164–172)	HLA-C*03:03	0.67	0.12	0.79	11.5	0.5056
			HLA-C*12:03				25.7	
			HLA-A*68:01				144.7	
			HLA-C*14:02				188.2	
			HLA-A*11:01				436.8	
6	AEELTEEGL	Thioredoxin family protein (287–295)	HLA-B*40:01	1.47	0.42	1.89	37.1	1.1538
			HLA-C*12:03				57.6	
			HLA-C*03:03				65.6	
			HLA-B*15:02				155.3	
			HLA-C*05:01				173.6	
7	LNSNERIEY	Thioredoxin family protein (249–257)	HLA-C*12:03	1.43	1.14	2.57	22.8	0.5401
			HLA-C*03:03				28.1	
			HLA-C*14:02				240.8	
			HLA-A*29:02				428.3	
			HLA-B*35:01				485.9	
8	LIAFGLVIL	ALT2 (5–13)	HLA-C*03:03	1.54	0.5	2.04	45.8	0.7180
			HLA-B*15:02				74	
			HLA-C*12:03				122.5	
			HLA-A*02:06				239.1	
			HLA-A*02:01				336.6	
9	RQGIGENVY	Venom allergen antigen-like protein 1 (93–101)	HLA-C*12:03	1.61	1.32	2.92	18.5	1.1047
			HLA-B*15:01				59.4	
			HLA-A*30:02				140.1	
			HLA-C*03:03				229.4	
			HLA-C*05:01				415.6	
10	KGKWLILFF	Peroxiredoxin 1 (61–69)	HLA-C*12:03	1.51	1.01	2.52	17.5	1.6196
			HLA-A*30:01				209.4	
			HLA-B*15:02				436.7	
11	ADDITICGF	Transglutaminase (154–162)	HLA-C*12:03	1.36	0.94	2.29	17.1	1.9539
			HLA-C*05:01				61.8	
			HLA-C*08:02				162.6	
			HLA-B*15:02				187.6	
			HLA-C*14:02				191.3	
12	ADTERDRFK	Transglutaminase (177–185)	HLA-C*03:03	0.82	0.14	0.96	21.3	0.8263
			HLA-C*12:03				48.3	
13	SNYWRNRVL	Transglutaminase (276–284)	HLA-C*03:03	1.64	0.44	2.08	13.1	2.2390
			HLA-B*15:02				30.2	
			HLA-C*12:03				50	
			HLA-C*14:02				55.2	
			HLA-C*15:02				66.7	
			HLA-C*07:01				69.4	
			HLA-C*06:02				172.5	
			HLA-B*14:02				392.7	
14	VLIEFYAPW	Transglutaminase (390–398)	HLA-C*12:03	0.98	0.39	1.36	27.6	0.9901
			HLA-C*03:03				32.9	
			HLA-C*14:02				119.8	
			HLA-B*58:01				158.2	
			HLA-A*32:01				173.4	
			HLA-A*02:01				189.3	
			HLA-A*02:06				384.2	
			HLA-B*57:01				484	
15	GREVDDFIK	Transglutaminase (463–471)	HLA-C*12:03	1.03	0.14	1.17	41.1	0.4956
			HLA-C*03:03				286.2	
			HLA-C*07:01				309.2	
			HLA-C*14:02				489.4

**Table 4:** T_H_ epitopes with its binding allele (IC50 < 500 nM)

S.no	HTL epitope	Position	Allele	IC50	Antigenicity (VaxiJen score)
1	GLFLIDPSGEIRHSL	Thioredoxin peroxidase (156–170)	HLA-DRB3*01:01	27	0.5924
			HLA-DRB1*03:01	179	
			HLA-DRB1*07:01	192	
			HLA-DRB1*01:01	263	
			HLA-DRB1*13:02	350	
			HLA-DRB5*01:01	403	
2	FNPILESNSNGTLNI	Cuticular glycoprotein gp29 (83–97)	HLA-DRB1*13:02	38	0.6509
			HLA-DRB1*01:01	212	
			HLA-DRB1*07:01	235	
			HLA-DRB1*04:01	259	
			HLA-DRB1*04:05	371	
			HLA-DRB1*04:04	471	
3	SSFCYIQETVRGSVD	Thioredoxin (8–22)	HLA-DRB5*01:01	40	0.4336
			HLA-DRB1*01:01	142	
			HLA-DRB1*07:01	181	
			HLA-DRB1*11:01	406	
			HLA-DRB1*04:01	448	

### Linear B cell epitope prediction

Totally 60 B cell epitopes from FBCPred and 222 epitopes from the ABCPred server were obtained. A total of 11 common peptides were scrutinized between those two servers’ results which were further checked for antigenicity, allergenicity, and toxicity. Finally, three highly antigenic LBL epitopes were found as shown in Table 5.

**Table 5:** B cell epitopes selected by FBCPred and ABCPred server

S.no	LBL epitopes	Position	Antigenicity (VaxiJen score)	Antigen/non-antigen	Allergenicity	Toxicity
1	FPCNQFYLQEPAENHE	Cuticular glycoprotein gp29 (100–116)	1.2474	ANTIGEN	NON-ALLERGEN	NON-TOXIN
2	LSENQAWTDRGCFCED	ALT2 protein (84–100)	0.9334	ANTIGEN	NON-ALLERGEN	NON-TOXIN
3	NPTGQVPVLIDNNFII	Glutathione S-transferase (44–60)	1.0852	ANTIGEN	NON-ALLERGEN	NON-TOXIN

### Multi-epitope subunit vaccine construction

**Fig. 2:**
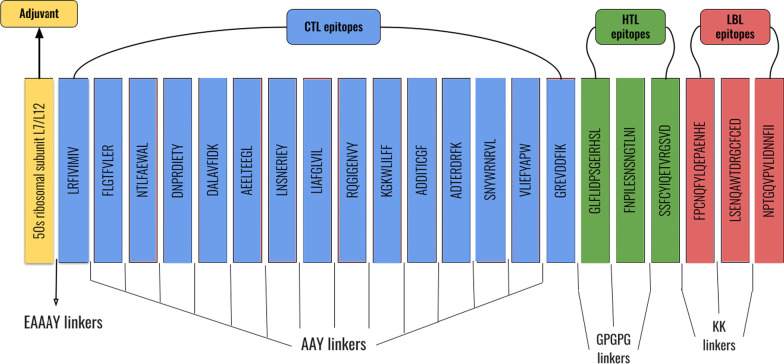
Diagrammatic representation of the final vaccine construct

The total number of predicted epitopes used in designing the vaccine was 15 CTL epitopes, 3 HTL epitopes, and 3 LBL epitopes. The predicted peptide sequences containing our CTL, HTL, and LBL epitopes were fused with the help of appropriate linkers (AAY, GPGPG, and KK). In order to enhance antigen-specific immune responses, 50S ribosomal L7/L12 was ligated to the amino terminus of the vaccine peptide via an EAAAK linker. Our final vaccine peptide comprised of 426 amino acid residues derived from 21 combined peptide sequences (Fig. 2).

### Evaluation of antigenicity and allergenicity of the designed vaccine candidates

The antigenicity of the final sequence (including the adjuvant) was assessed to be 0.5876 by the VaxiJen 2.0 server with a parasitic model at a threshold of 0.4. The main vaccine sequence (without adjuvant) gave a score of 0.7245 with the parasite model at a threshold of 0.4 on VaxiJen 2.0. The results show that the predicted vaccine sequences (with and without adjuvant) are both antigenic in nature. On the AllerTOP v.2 server, the vaccine sequence with and without adjuvant was predicted to be non-allergenic as well.

### Physiochemical properties and solubility prediction

The different physicochemical properties of our final vaccine construct are presented in Table 6. The molecular weight of the vaccine construct was calculated to be 46.39 kDa with the theoretical pI 4.67 which corroborated the acidic nature. The vaccine’s stability was represented by the instability index of 26.34 (a score of less than 40 indicates a stable protein). The half-life of mammalian reticulocytes (in vitro) was evaluated to be 30 h, yeast (in vivo) to be > 20 h, and *Escherichia coli* to be > 10 h (in vivo). The aliphatic index of 92.49 indicates thermostability, while the GRAVY rating of 0.027 indicates the very slight hydrophobic nature of our vaccine. The construct was also shown to be soluble in the ProteinSol server (Additional file 4: Fig. S1).

**Table 6:** Physiochemical properties identification of our vaccine

Number of amino acids	426
Molecular weight	46,392.74
Theoretical pI	4.67
Formula	C_2119_H_3248_N_528_O_626_S_8_
Total number of atoms	6529
Aliphatic index	92.49
Grand average of hydropathicity (GRAVY)	0.027
Estimated half-life	30 h (mammalian reticulocytes, in vitro) > 20 h (yeast, in vivo) > 10 h (*Escherichia coli*, in vivo)
Instability index	26.34

### Cross-reactivity between designed vaccine candidate and human proteome

The shortlisted epitopes were subjected to NCBI-BLAST for searching similarities against the human proteome; however, no significant matches were found. This shows that our predicted epitopes may not cause cross-reactivity reactions with the human proteome.

### Population coverage analysis of CTL and HTL epitopes

**Fig. 3:**
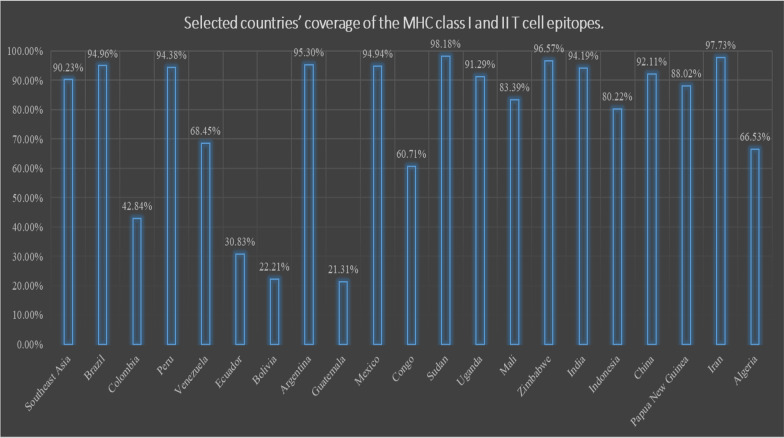
Population coverage of T cell epitopes with their HLA alleles against the hotspots of LF

HLA allelic distribution varies by geographical region and ethnic group around the world. As a result, in addition to developing an effective vaccine, population coverage must be considered. HLA alleles relevant to our chosen CTL and HTL epitopes were collected individually and merged for population study against the hotspot regions of LF**.** The strongest continental coverage was found in South-East Asia (Fig. 3), and this region is reported to have a high prevalence of *Brugia malayi* cases. The study revealed that our vaccines represent 94.96%, 94.38%, 95.30%, 94.94%, 98.18%, 91.29%, 96.57%, 94.19%, 92.11%, and 97.73% coverage of the HLA alleles in Brazil, Peru, Argentina, Mexico, Sudan, Uganda, Zimbabwe, India, China, and Iran populations, respectively.

### Secondary structure prediction

**Fig. 4:**
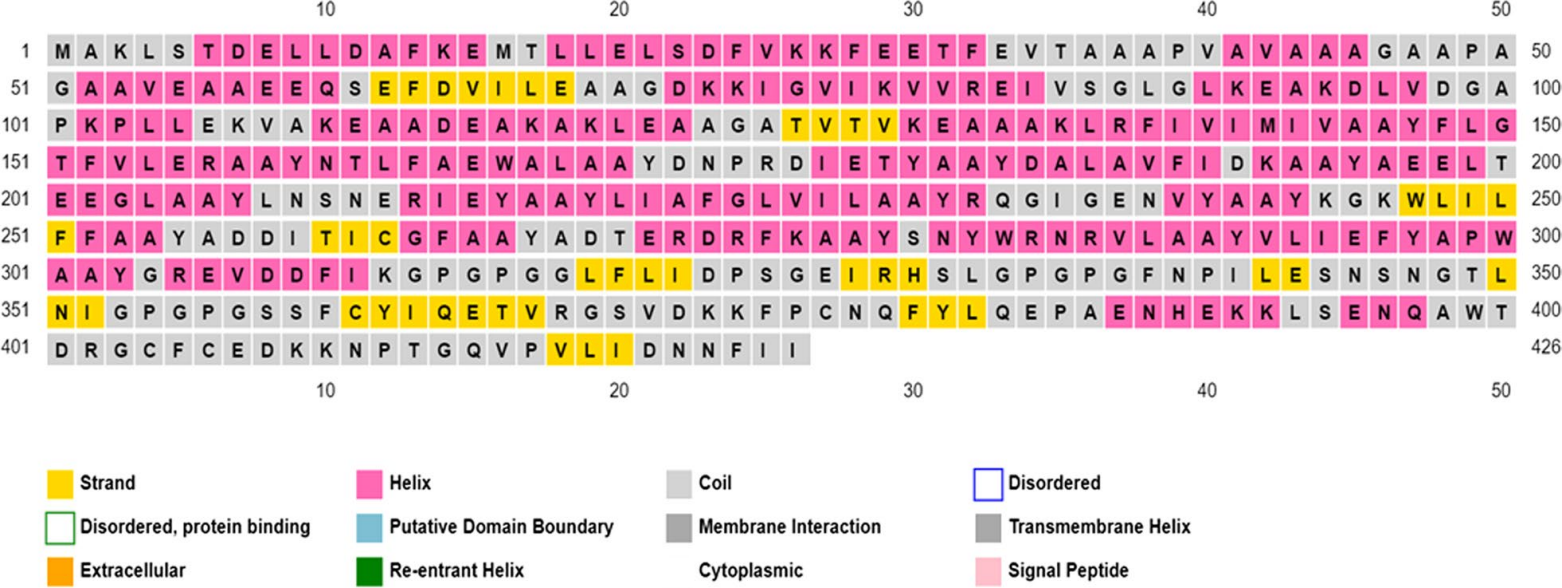
Secondary structural predictions of the vaccine sequence

The secondary structure composition of our vaccine model can be majorly classified as helix, coil, and strand regions. Figure 4 obtained from the PSIPRED server represents a graphical illustration of the various regions in which almost 53% (227 residues) accounted for a helix, 10% (44 residues) accounted for b strands and 36% (155 residues) accounted for random coils.

### Tertiary structure modelling, refinement, and validation

**Fig. 5:**
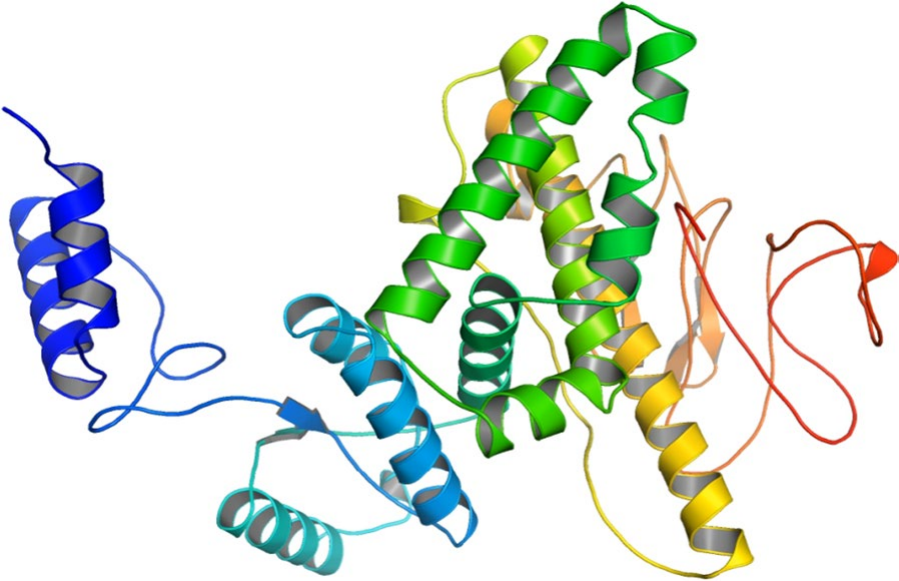
A cartoon representation of the tertiary structure of the vaccine construct

**Fig. 6:**
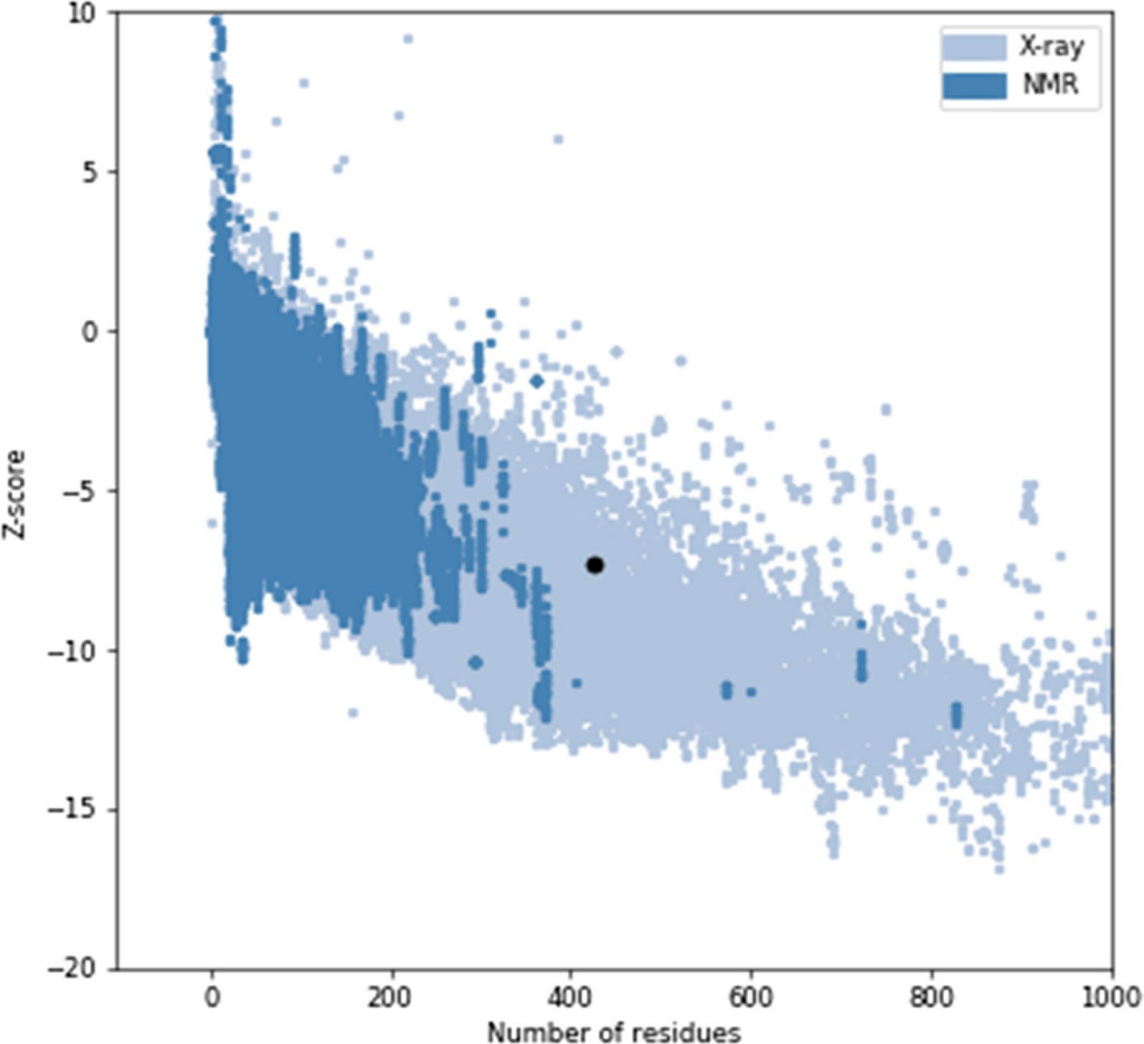
Tertiary structural validation by ProSA

**Fig. 7:**
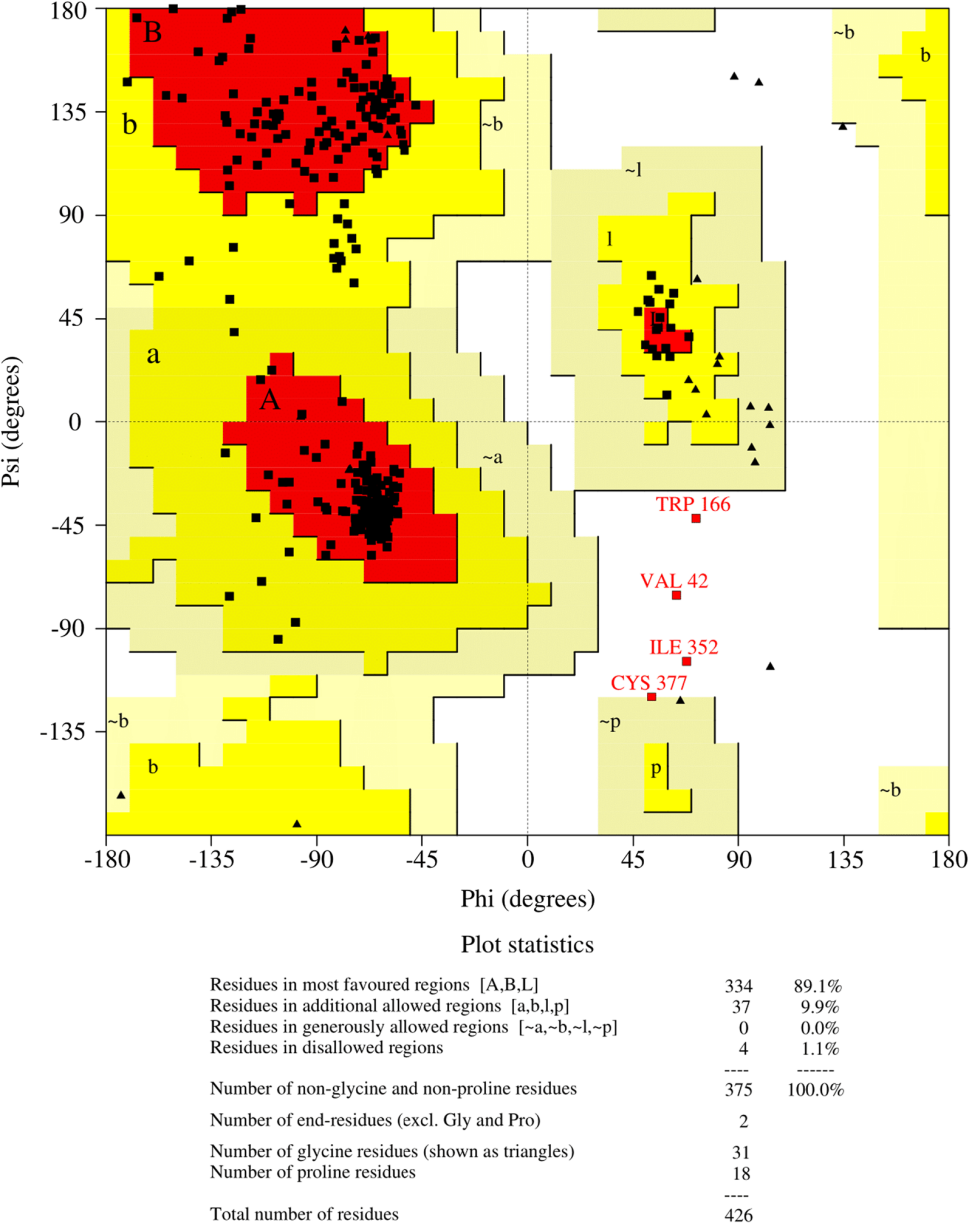
Ramachandran plot obtained from PROCHECK

The tertiary structure of our vaccine model was predicted through RaptorX and refined with the GalaxyRefine server. Figure 5 depicts a cartoon representation of the tertiary structure for our vaccine. The energy minimization of the refined model was done through a Swiss-PDB viewer, and the final structure was validated using ProSA and PROCHECK. The Z-score value obtained from ProSA is − 7.34, and Fig. 6 shows a graphical illustration wherein the Z-score lies within the acceptable range for a residue length of 426. Figure 7 depicts the Ramachandran plot that was obtained from PROCHECK in which 89.1% of the residues lie in the most favoured regions and 9.9% of the residues lie in the additionally allowed regions.

### Conformational B cell epitope identification of final vaccine

A total of 14 discontinuous B cell epitopes were identified in Ellipro which crossed the threshold score of 0.5. The length of the epitopes had a broad range of 5 to 44 residues, and Table 7 represents the start and end positions of the respective epitopes in the overall sequence.

**Table 7:** List of conformational B cell epitopes obtained from Ellipro

S. No	Start position	End position	Peptide sequence	No. of residues	Score
1	1	44	MAKLSTDELLDAFKEMTLLELSDFVKKFEETFEVTAAAPVAVAA	44	0.877
2	264	275	FAAYADTERDRF	12	0.758
3	160	181	NTLFAEWALAAYDNPRDIETYA	22	0.757
4	103	125	PLLEKVAKEAADEAKAKLEAAGA	23	0.732
5	48	61	APAGAAVEAAEEQS	14	0.695
6	384	403	EPAENHEKKLSENQAWTDRG	20	0.679
7	332	357	LGPGPGFNPILESNSNGTLNIGPGPG	26	0.673
8	407	416	EDKKNPTGQV	10	0.665
9	312	317	KGPGPG	6	0.648
10	367	375	VRGSVDKKF	9	0.642
11	229	239	AAYRQGIGENV	11	0.638
12	198	202	ELTEE	5	0.606
13	302	309	AYGREVDD	8	0.6
14	67	72	LEAAGD	6	0.502

### Molecular docking analysis and interaction studies

**Fig. 8:**
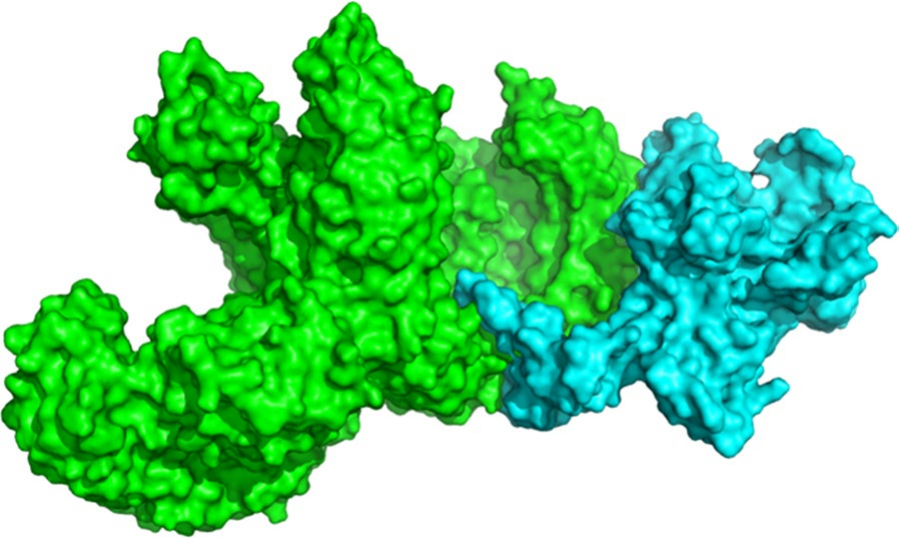
A docked cluster of our vaccine with TLR4 receptor (vaccine model in cyans and TLR4 receptor in greens)

A total of 21 docked protein complexes were obtained from the ClusPro server each having a representative centre weighted score and the lowest energy weighted score. Among the 21 clusters, the 13th cluster was having the lowest energy score of − 969 and hence it was selected as the best complex. It is also notable that the 13th cluster was the only cluster in which the centre weighted score was the same as the lowest energy weighted score (− 969 for both), thus re-affirming our selection. Figure 8 shows a surface representation of the docked protein complexes. The non-bonded interactions between the two proteins that were further analysed in the Discovery studio visualizer revealed that a total of 24 residues are involved in a number of hydrogen bonds, salt bridges, electrostatic bonds, and hydrophobic interactions. It is believed that all these interactions act synergistically to produce an overall effect in the binding complex. Table 8 represents the type of interaction specific to particular residues.

**Table 8:** List of non-bonded interactions at the surface of docking between our vaccine model and TLR4 receptor

Interaction category				
Salt bridges	Hydrogen bonds	Electrostatic bonds	Hydrophobic bonds	Total no. of interacting residues
ARG355 and GLU336	TYR72, GLN99, GLY147, LYS150, ASN309, SER312, ARG355 (3), GLU287 (2), HIS256, SER127, SER126, GLN99, SER123 (2), GLU142, HIS148, GLU169, SER172 and GLY96	ARG355, ASP95, LYS150 and GLU286	HIS334, TYR72 (2), HIS148, HIS256 and ILE285	24

### Normal mode analysis

**Fig. 9:**
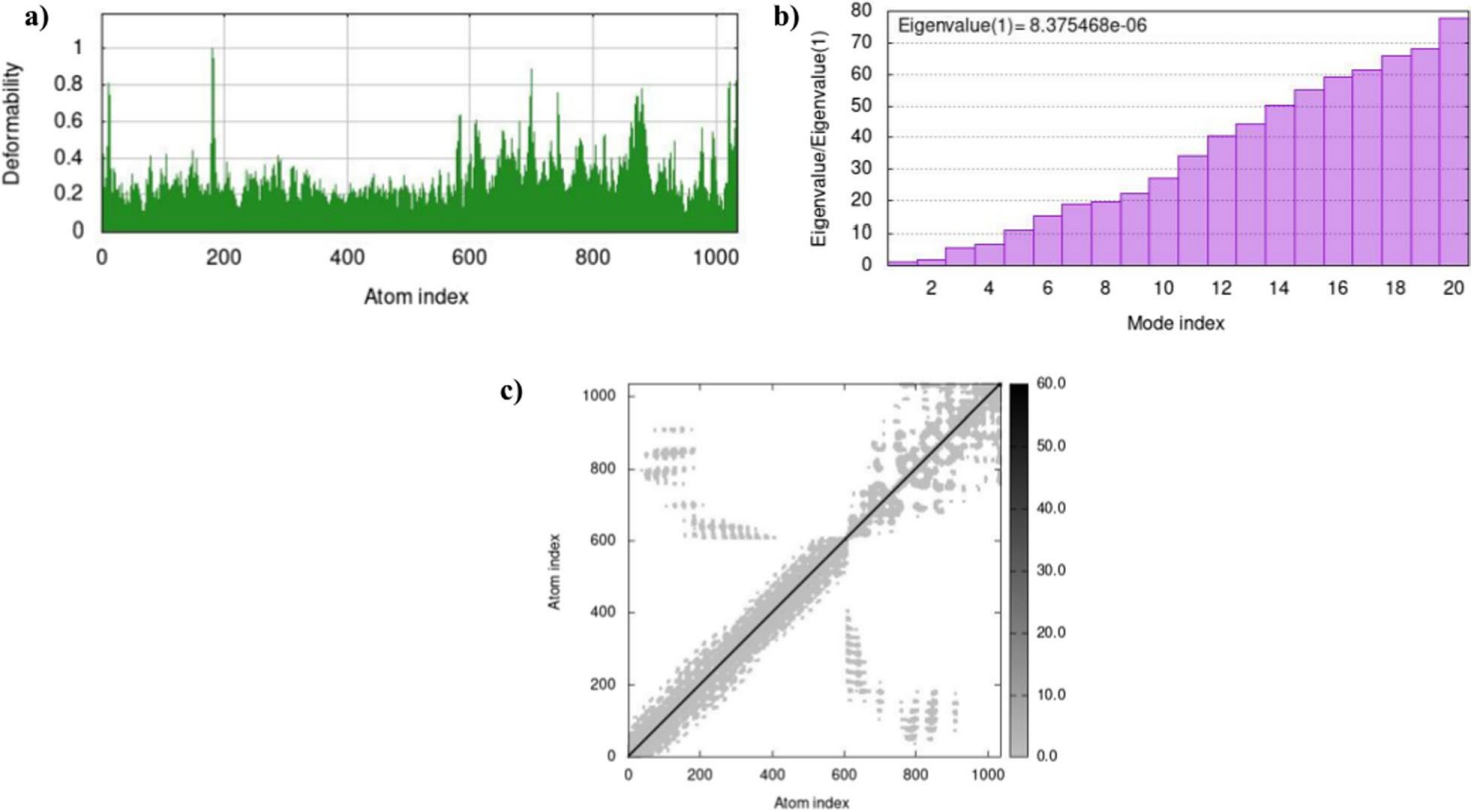
**a** Deformability, **b** Eigenvalue, and **c** Elastic network of our constructed vaccine protein model

The results of the normal mode analysis obtained from the iMODS server provided information on the conformational stability and biophysical alterations of the vaccine-receptor complex. The deformability plot shown in Fig. 9a indicates the non-rigid portions as peaks nearing the value of 1, meaning that those atoms are prone to fluctuations and thereby rendering flexibility to the protein. Figure 9b represents the eigenvalue of the docked complex which is directly related to the lowest energy required to deform the complex. The eigenvalue for our protein was 8.375468e-06 which is a measure of the overall stiffness of the protein complex. Figure 9c shows an elastic network graph that represents the pair of atoms connected by springs and each dot in the graph indicates one spring between the corresponding pair of atoms. In the graph, a darker grey represents a stiffer spring between the atoms.

### Immune simulation

**Fig. 10:**
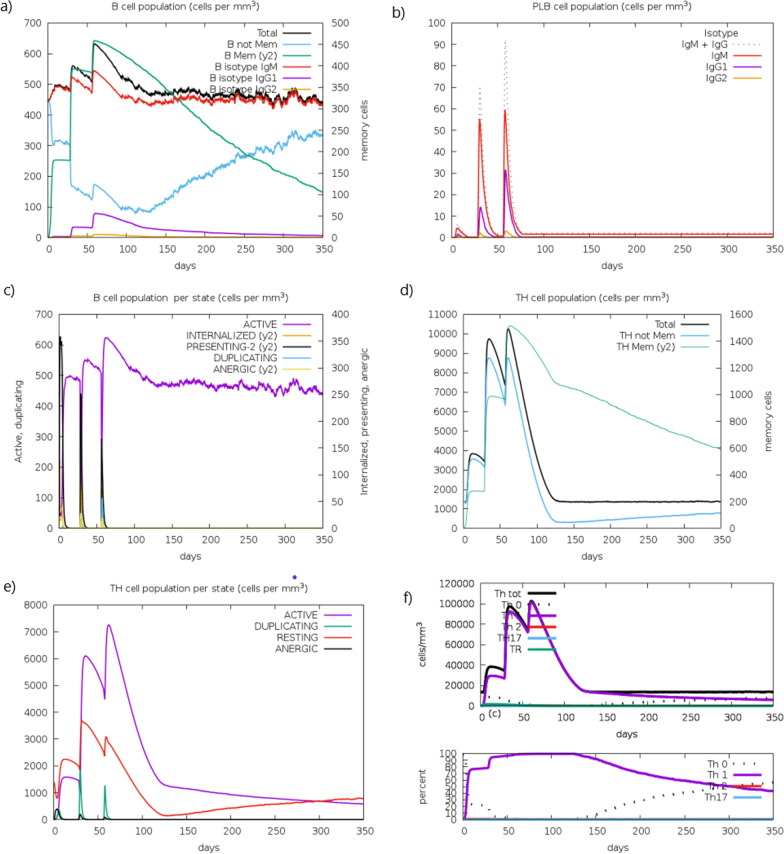
**a** B cell population **b** PLB cell population **c** B cell population per state **d** TH cell population **e** TH cell population per state **f** T regulatory cells population

**Fig. 11:**
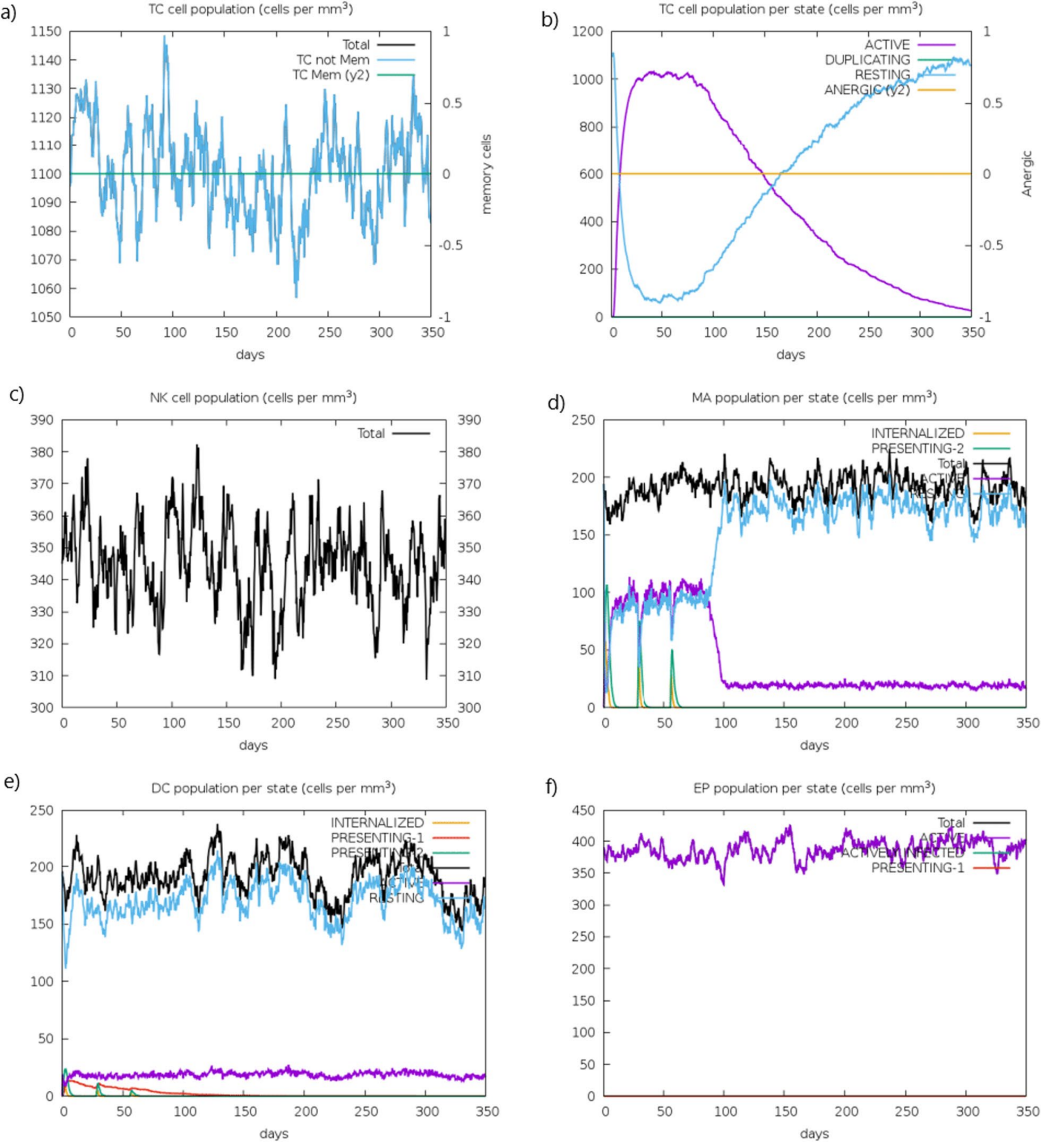
**a** TC cell population **b** TC cell population per state **c** Natural killer cell population **d** Macrophages population per state **e** Dendritic cell population per state **f** Epithelial cell population per state

**Fig. 12:**
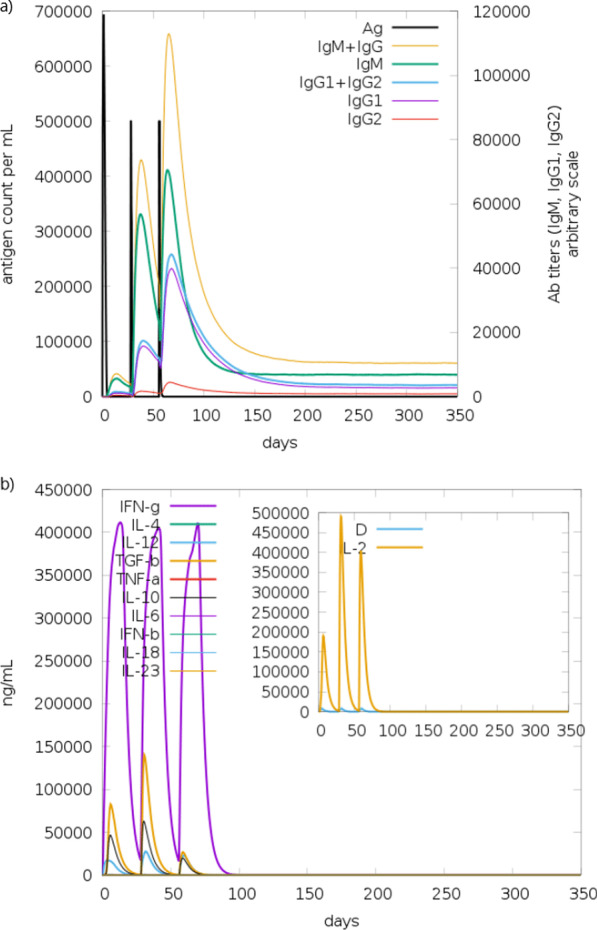
**a** Production of several subclasses of immunoglobulin (coloured lines) in response to vaccine injection (black vertical lines) **b** Production of Cytokine and Interleukin

The results of the immune simulation studies obtained from the C-ImmSim server revealed that our vaccine is capable of inducing an effective immune response through a number of mechanisms including but not limited to an increased B cell, T cell and NK cell population. A relatively high amount of IgM was observed than the immunoglobulin IgG. There was a concurrent decrease in the antigen levels followed by an enhanced level of the IgG1 + IgG2, IgM, and IgG + IgM antibodies during the secondary and tertiary responses. An increased antigen clearance was observed in the subsequent exposures owing to the development of immune memory. Our vaccine was able to provide effective and long-lasting protection by generating increased levels of activated memory B cells. In addition, the helper T cells, cytotoxic T cells, and regulatory T cells also showed a strong response to immune memory. The dendritic cells, natural killer cells, and macrophages worked in unison to complement each of their immune responses. Furthermore, the innate immune system components like epithelial cells were also involved. Overall, there was an appreciable immune response to our vaccine especially after the second and third titres as it is demonstrated by the graphical illustrations in Figs. 10, 11 and 12.

### Codon optimization, mRNA structure prediction, and in silico gene cloning

In order to prove the expression efficiency of our vaccine, in silico gene cloning was performed in a suitable expression vector. For this purpose, the amino acid sequence of our vaccine was subjected to reverse transcription using the Java codon adaptation tool and thereby the codons were optimized. After codon optimization, the length of the entire nucleotide sequence was 1278. The average GC content of the optimized nucleotide sequence was 50.54%, while the ideal range of GC content for any nucleotide sequence should be between 30 and 70% for it to be thermodynamically stable. The CAI value of the improved sequence was 0.96 and hence falling within the acceptable range.

The optimal secondary structure of mRNA had a minimum free energy of − 372.10 kcal/mol. The centroid structure had a minimum free energy of − 278.07 kcal/mol, and the free energy of the thermodynamic ensemble was − 393.80 kcal/mol. Additional file 5: Fig. S2 represents the minimum free energy structure encoding its positional entropy, and Additional file 6: Fig. S3 represents the centroid structure. Additional file 7: Fig. S4 shows a mountain plot of the minimum free energy structure, the thermodynamic ensemble of RNA structures, and the centroid structure along with a graphical illustration of the positional entropy at each position.

**Fig. 13:**
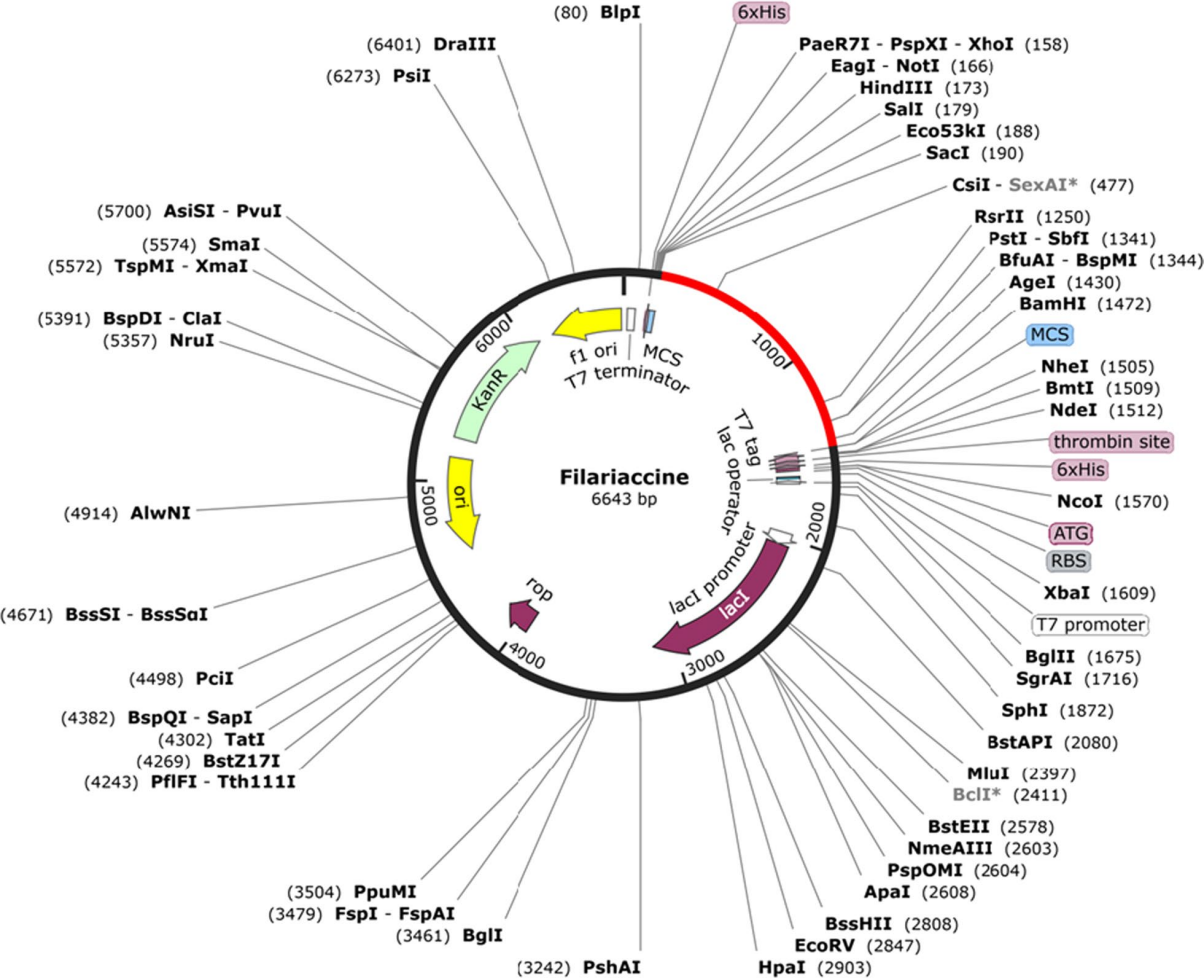
A diagrammatic map of in silico gene cloning

Finally, our improved gene sequence was cloned into the vector pET28a (+) by introducing the restriction enzymes EcoRI and BamHI at the N terminal and C terminal positions of our vaccine’s nucleotide sequence, thereby forming a recombinant plasmid in Fig. 13.

## Discussion

Although lymphatic filariasis caused by Brugian species is responsible for only 10% of the global LF infections, it still contributes to a huge disease burden in South-east Asian countries (Chandrasena et al. 2019). It parasitizes human lymphatics, causing lymphostasis, hydrocele, lymphedema, and, eventually, elephantiasis. The medications approved by the FDA are only effective against microfilaria and have no effect on adult worms. Recently, attention has shifted to the development of peptide vaccines, which have excellent safety profiles and are logistically more feasible. Epitope-based vaccines are a novel strategy for eliciting a specific immune response while avoiding reactions to other unfavourable epitopes (such as epitopes that may cause immunopathogenic or immunological modifying responses) in the complete antigen.

In this study, several in silico assessments were therefore used to design and develop a multi-epitope peptide-based vaccine for LF by screening eight highly antigenic proteins of *Brugia malayi*, obtained through a literature review. So, in order to develop both humoral and cytotoxic immune responses, the CTL, HTL, and LBL epitopes were first identified from the shortlisted antigenic proteins by the various immunoinformatics tools. Then, using specific servers stated in the methodology section, the best epitopes were chosen for vaccine construction that proved antigenicity, non-allergenicity, non-toxicity, and immunogenicity.

Finally, the CTL, HTL, and LBL epitopes were connected with vaccine compatible-peptide liners (AYY, GPGPG, KK) to construct the final vaccine. It is noteworthy that the GPGPG and AAY linkers aid in generating controlled functional immunogenicity, while the EAAAK is found to segregate domains of functional protein structure. Additionally, to boost the immunogenicity, longevity, and most importantly, the vaccine construct’s stability, an adjuvant—50S ribosomal protein L7/L12 which is an agonist of human TLR4, was added at the N terminal position of the vaccine. Several prediction scores from various web tools made it possible to design an antigenic as well as the non-allergenic vaccine. Adjuvant interactions with TLR4 polarize cytotoxic T cell responses and generate robust immunological responses. Furthermore, the population coverage analysis demonstrated that the shortlisted epitopes would be excellent vaccine candidates for people of South-east Asian countries as well as the other hotspots of LF.

An effective vaccine should have good physicochemical characteristics during production, formulation, consumption, and storage. The ExPASy ProtParam server predicted the vaccines’ physicochemical properties. Physicochemical properties include the number of amino acids, molecular weight, theoretical isoelectric point (pI), formula, the total number of atoms, aliphatic index, GRAVY, estimated half-life and instability index. The vaccines’ molecular weight would be utilized as an indication in SDS-PAGE electrophoresis and Western blot analysis. The pH at which a molecule has zero net electrical charge is known as theoretical pI. The vaccine’s theoretical pI was calculated to be 4.67, indicating that it was acidic in nature and the aliphatic index of 92.49 confirmed its thermostability which means the approximate occupied mass by aliphatic side chains of the vaccine (Table 6). The half-life period obtained from the server is related to the time of disappearance after the vaccine’s administration. The instability index of our vaccine being lower than 40 indicates that the vaccine is classified as stable (Table 6). GRAVY is an indication of the average hydropathy values of amino acids in a protein. Protein solubility is a critical feature in predicting a vaccine’s immunogenicity. Since insoluble proteins are non-immunogenic, solubility index was checked by the ProteinSol server, where it proved its effective interaction with water (Additional file 4: Fig. S1). The constructed vaccine ensured that it could be easily expressed inside the host cell of E. coli due to its solubility and predicted half-life of > 10 h in vivo (Table 6).

The secondary structural composition of the vaccine sequence shows that nearly half of the sequences would form a helices (53%), followed by random coils (36%) and only 10% of the residues would form b strands. The confidence level of prediction by the PSIPRED server was also pretty high for most of the residues (Buchan and Jones 2019). The few discrepancies in the tertiary structure predicted through the RaptorX software were significantly improved after refinement by the GalaxyRefine server. There was also a slight increase in the Ramachandran plot favoured residues (from 96.5 to 96.9) post-refinement. The Z-score graph obtained from ProSA provides a surface range for X-ray crystallographic structures and NMR spectroscopy structures for appropriate residue lengths. Though the Z-score − 7.34 outlies the region for NMR, it falls well within the range for X-ray crystallographic structures. The knowledge-based energy graph validates the thermodynamic stability of the protein since there are very few peaks crossing the positive energy axis. Generally, for a valid protein structure in the Ramachandran plot, the percentage of residues in the allowed regions should be greater than 90%. For our constructed vaccine, it was 99% (including most favoured, additionally allowed, and generously allowed regions) and hence being strongly validated.

Following the validation of the vaccine’s tertiary structure, the discontinuous B cell epitopes were predicted using the Ellipro tool. Among the 14 epitopes that crossed the threshold value of 0.5, an epitope of length 44 residues had a maximum score of 0.877, an epitope of length 6 residues had a minimum score of 0.502, 3 of the epitopes had a moderate score of above 0.7 and the remaining 9 epitopes had their scores around 0.6. The docked cluster obtained from the ClusPro tool depicted our vaccine model being strongly buried within the cavities of the TLR4 receptor (Kozakov et al. 2017). In addition, several non-bonded interactions are known to be occurring between the two protein complexes that complement their effective binding and interaction (Yuan et al. 2017). During the normal mode analysis, the flexibility, stiffness, and overall biophysical stability of our vaccine were strongly validated by the deformability graph, eigenvalue plot as well as elastic network map, respectively.

Though the structure and stability of our vaccine model were strongly validated in the preliminary studies, the ability of the protein to induce an appropriate immune response remained a sceptical question until it was proven by the immune simulation studies that were performed through the C-ImmSim tool. The population of various immune cells such as the immunoglobulins (IgG, IgM, IgG + IgM), TH cells, TC cells, natural killer cells, macrophages, dendritic cells, and even epithelial cells were critically analysed during various states. Since the simulation was run for 12 months (1050) with the regular administration of 3 titres on day 1 (1st-time step), day 28 (84th-time step), day 56 (168th-time step), the B cell population, as well as the T cell population, saw a significant rise after the second and third titres. The antigen clearance pattern followed an inverse correlation to the levels of memory cells and thereby producing a long-lasting protective immunity.

Previous research efforts have demonstrated that IgG1 and IgG3 antibodies in the peripheral circulation of the infected patients kill *Brugia malayi* infective larvae in an ADCC reaction. It has been shown that these cytophilic antibodies in humans correlate with the absence of infection. The development of prophylactic vaccines based on antigens or peptides that induce these protective antibodies is therefore of interest (Morris et al. 2013). Furthermore, the elevated level of IgE follows a consistent pattern in filarial infections. In order to minimize any adverse effects, the selected antigens should not be homologous or have little homology to human proteins and should not elicit any IgE or IgG4 responses. Considering these points, our vaccine produced appreciable levels of IgG1 antibodies and demonstrated zero levels for IgE or IgG4 antibody production. Hence, our vaccine would be capable of killing the *Brugia malayi* larvae during the infective stages. Also, the vaccine’s sequence showed no significant homology with the human proteome when checked for cross-reactivity using the BLASTP tool. This adds to another advantage of our vaccine being non-allergenic to humans.

The protein sequence of the vaccine was reverse transcribed to nucleotide sequence and optimized to improve its expression levels using the Java codon adaptation tool. The optimized nucleotide sequence had an ideal value of CAI index as well as an ideal percentage of GC content which are crucial parameters for the expression and stability of the gene. The minimum free energy and the positional entropy of the predicted mRNA structure rendered an outline about the thermodynamic stability of the mRNA during the transcription process. The optimized nucleotide sequence was finally cloned into a recombinant plasmid vector pET28a (+). The insertion was facilitated by the two popular restriction enzymes EcoRI and BamHI at the N terminal and C terminal positions, respectively (Gorai et al. 2022).

## Conclusion

Among the several debilitating infections affecting the human host, the neglected tropical diseases need immediate attention for the reason that these diseases often affect the marginalized community of citizens who seldom have access to high-end medical facilities and treatments. A sustainable cure for such NTDs would be the development of effective vaccines that would not only provide long-lasting protective immunity, but also aid in the complete eradication of the disease in the long run. Besides the various other treatment strategies like mass drug administration programmes and safe sanitation practices, a prophylactic vaccine would be of great help to control the rapid spread of the infection. Here in our research, we adopted a reverse vaccinology approach to construct and validate a multi-epitope-based vaccine model for lymphatic filariasis, by considering the antigenic proteins from the target organism *Brugia malayi*. We believe that our designed vaccine model—“Filariaccine”, has passed through a remarkably high number of validation studies, proving its thermodynamic stability, effective binding as well as ability to induce a strong immune response in the host. The total 21 HTL, CTL, and LBL epitopes that were carefully selected after checking their antigenicity, immunogenicity, allergenicity, and toxicity properties were also found to be matching the HLA alleles of the entire world population, specifically in the hotspots of NTDs. The cross-reactivity analysis revealed that our vaccine does not overlap with the human proteome and hence bringing a great advantage. Despite having strong evidence for the effectiveness of our vaccine, relevant in vitro and in vivo studies are mandatory before subjecting the synthetic vaccine to clinical trials, which are also the future directions of this research. Our constructed in silico vaccine model would be a key towards understanding the essential properties of the vaccine, let alone serving as a preliminary design. Provided that our vaccine surpasses all the animal and human trials, this would be of immense help to LF patients across the globe.

## Supplementary Information

**Additional file 1: Table S1.** The list of 17 neglected tropical diseases as recognized by WHO with their causative organisms and mode of transmission.

**Additional file 2: Table S2.** List of 49 frequently occurring MHC-I binding alleles.

**Additional file 3: Table S3.** List of 15 frequently occurring MHC-II binding alleles.

**Additional file 4: Fig. S1.** The solubility of the vaccine (QuerySol) predicted against the average solubility of the Escherichia coli protein (PopAvrSol).

**Additional file 5: Fig. S2.** Minimum free energy structure of mRNA encoding its position entropy.

**Additional file 6: Fig. S3.** Centroid structure of mRNA encoding its position entropy.

**Additional file 7: Fig. S4.** A mountain plot of minimum free energy structure, the thermodynamic ensemble of RNA structures, and the centroid structure along with a graphical illustration of the positional entropy at each position.

## Data Availability

All necessary data generated or analysed during this study are included in this article. Any additional data could be available from the corresponding author upon request.

## Abbreviations

ACC: Auto-cross-covariance
ALT: Abundant larval transcript
BLAST: Basic local alignment search tool
CAI: Codon adaptation index
CTL: Cytotoxic T lymphocytes
FAB: Embryonic fatty acid-binding protein
GDT-HA: Global distance test-high accuracy
GRAVY: Grand average of hydropathicity
GST: Glutathione S-transferase
HLA: Human leukocyte antigens
HTL: Helper T lymphocytes
IFN: Interferon
IL: Interleukin
JCat: Java Codon Adaptation Tool
LBL: Linear B cell lymphocytes
LF: Lymphatic filariasis
MDA: Mass drug administration
MHC: Major histocompatibility complex
NK cells: Natural killer cells
NMA: Normal mode analysis
NTD: Neglected tropical diseases
PI: Protrusion index
PSI-BLAST: Position-specific iterated-basic local alignment search tool
PSSM: Position-specific scoring matrix
RMSD: Root-mean-square deviation
SMM: Stabilized matrix method
TAS: Transmission assessment surveys
TLR: Toll-like receptor
TPX: Thioredoxin peroxidase
VAH: Venom allergen antigen-like protein 1
WHO: World Health Organization
