# Supplementary material for: Construction and validation of a multi-epitope in silico vaccine model for lymphatic filariasis by targeting Brugia malayi: a reverse vaccinology approach
Source: Bull Natl Res Cent. 2023 Mar 24;47(1):47. doi: 10.1186/s42269-023-01013-0 (PMC10037386; doi:10.1186/s42269-023-01013-0)
Supplement: Supplementary file 1 — Additional file 1: Table S1. The list of 17 neglected tropical diseases as recognized by WHO with their causative organisms and mode of transmission. [file 42269_2023_1013_MOESM1_ESM.docx]

**Supplementary table 1.** The list of 17 neglected tropical diseases as recognized by WHO with their causative organisms and mode of transmission.

| **Category of infection** | **S.No** | **Neglected Tropical disease** | **Causative organisms** | **Mode of transmission** |
| --- | --- | --- | --- | --- |
| Protozoan infections | 1 | Chagas disease | Trypanosoma cruzi | Insect vectors (Kissing bugs) |
|  | 2 | Human African trypanosomiasis | Trypanosoma brucei | Tse Tse flies |
|  | 3 | Leishmaniasis | Leishmania parasites | Phlebotomine sandflies |
| Helminth infections | 4 | Taeniasis | Taenia saginata (beef tapeworm), Taenia solium (pork tapeworm), and Taenia asiatica (Asian tapeworm) | Raw or undercooked meat |
|  | 5 | Dracunculiasis | Dracunculus medinensis | Drinking water containing guinea worm larvae |
|  | 6 | Echinococcus | Echinococcus granulosus and Echinococcus multilocularis | Contaminated food and water |
|  | 7 | Foodborne trematodiases | Clonorchis, Opisthorchis, Fasciola and Paragonimus | Contaminated food |
|  | 8 | Lymphatic filariasis | Wuchereria bancrofti, Brugia malayi, and Brugia timori | Mosquitoes |
|  | 9 | Onchocerciasis | Onchocerca volvulus | Blackflies |
|  | 10 | Schistosomiasis | Blood flukes of the genus Schistosoma | Freshwater snails containing the infective larvae |
|  | 11 | Soil transmitted helminthiases (Ascariasis, hookworm diseases, trichuriasis, strongyloidiasis) | Ascaris lumbricoide (roundworm), Trichuris trichiura (whipworm), Necator americanus and Ancylostoma duodenale (hookworms) and Strongyloides stercoralis | Faeco-oral route |
| Bacterial infections | 12 | Buruli ulcer | Mycobacterium ulcerans | Contaminated water |
|  | 13 | Leprosy | Mycobacterium leprae | Aerosol spread of nasal secretions |
|  | 14 | Trachoma | Chlamydia trachomatis | Personal contact with infected patients |
|  | 15 | Yaws | Treponema pallidum | person-to-person contact of minor injuries |
| Viral infections | 16 | Dengue | Dengue virus(DENV) | Mosquitoes |
|  | 17 | Rabies | Rabies virus | Bite of infected animals |
