# Supplementary material for: Construction and validation of a multi-epitope in silico vaccine model for lymphatic filariasis by targeting Brugia malayi: a reverse vaccinology approach
Source: Bull Natl Res Cent. 2023 Mar 24;47(1):47. doi: 10.1186/s42269-023-01013-0 (PMC10037386; doi:10.1186/s42269-023-01013-0)
Supplement: Supplementary file 2 — Additional file 2: Table S2. List of 49 frequently occurring MHC-I binding alleles. [file 42269_2023_1013_MOESM2_ESM.docx]

**Supplementary table 2.** List of 49 frequently occurring MHC-I binding alleles.

| **MHC-I alleles** |
| --- |
| HLA-A*01:01 |
| HLA-A*02:01 |
| HLA-A*02:06 |
| HLA-A*03:01 |
| HLA-A*11:01 |
| HLA-A*23:01 |
| HLA-A*24:02 |
| HLA-A*25:01 |
| HLA-A*26:01 |
| HLA-A*29:02 |
| HLA-A*30:01 |
| HLA-A*30:02 |
| HLA-A*31:01 |
| HLA-A*32:01 |
| HLA-A*68:01 |
| HLA-A*68:02 |
| HLA-B*07:02 |
| HLA-B*08:01 |
| HLA-B*14:02 |
| HLA-B*15:01 |
| HLA-B*15:02 |
| HLA-B*18:01 |
| HLA-B*27:05 |
| HLA-B*35:01 |
| HLA-B*35:03 |
| HLA-B*38:01 |
| HLA-B*39:01 |
| HLA-B*40:01 |
| HLA-B*40:02 |
| HLA-B*44:02 |
| HLA-B*44:03 |
| HLA-B*46:01 |
| HLA-B*48:01 |
| HLA-B*51:01 |
| HLA-B*53:01 |
| HLA-B*57:01 |
| HLA-B*58:01 |
| HLA-B*58:02 |
| HLA-C*03:03 |
| HLA-C*04:01 |
| HLA-C*05:01 |
| HLA-C*06:02 |
| HLA-C*07:01 |
| HLA-C*08:02 |
| HLA-C*12:03 |
| HLA-C*14:02 |
| HLA-C*15:02 |
| HLA-E*01:01 |
| HLA-E*01:03 |
