# Supplementary material for: Construction and validation of a multi-epitope in silico vaccine model for lymphatic filariasis by targeting Brugia malayi: a reverse vaccinology approach
Source: Bull Natl Res Cent. 2023 Mar 24;47(1):47. doi: 10.1186/s42269-023-01013-0 (PMC10037386; doi:10.1186/s42269-023-01013-0)
Supplement: Supplementary file 3 — Additional file 3: Table S3. List of 15 frequently occurring MHC-II binding alleles. [file 42269_2023_1013_MOESM3_ESM.docx]

**Supplementary table 3.** List of 15 frequently occurring MHC-II binding alleles.

| **MHC-II alleles** |
| --- |
| HLA-DRB1*07:01 |
| HLA-DRB1*01:01 |
| HLA-DRB1*04:04 |
| HLA-DRB5*01:01 |
| HLA-DRB3*01:01 |
| HLA-DRB1*04:05 |
| HLA-DRB4*01:01 |
| HLA-DRB1*15:01 |
| HLA-DRB1*13:02 |
| HLA-DRB1*09:01 |
| HLA-DRB1*04:01 |
| HLA-DRB1*11:01 |
| HLA-DRB1*03:01 |
| HLA-DRB1*12:01 |
| HLA-DRB1*08:02 |
