# Supplementary material for: Construction and validation of a multi-epitope in silico vaccine model for lymphatic filariasis by targeting Brugia malayi: a reverse vaccinology approach
Source: Bull Natl Res Cent. 2023 Mar 24;47(1):47. doi: 10.1186/s42269-023-01013-0 (PMC10037386; doi:10.1186/s42269-023-01013-0)

**Supplementary figure 1.** The solubility of the vaccine (QuerySol) predicted against the average solubility of the Escherichia coli protein (PopAvrSol).


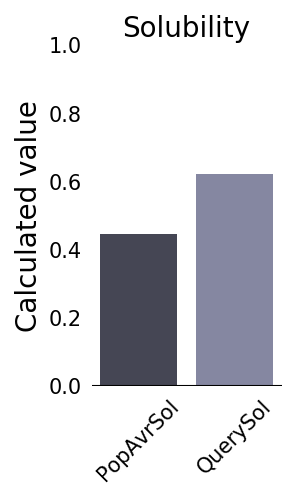

Supplement: Supplementary file 4 — Additional file 4: Fig. S1. The solubility of the vaccine (QuerySol) predicted against the average solubility of the Escherichia coli protein (PopAvrSol). [file 42269_2023_1013_MOESM4_ESM.docx]
