# Supplementary figures and images for: Construction and validation of a multi-epitope in silico vaccine model for lymphatic filariasis by targeting Brugia malayi: a reverse vaccinology approach
Source: Bull Natl Res Cent. 2023 Mar 24;47(1):47. doi: 10.1186/s42269-023-01013-0 (PMC10037386; doi:10.1186/s42269-023-01013-0)

**Supplementary figure 2.** Minimum free energy structure of mRNA encoding its position entropy.


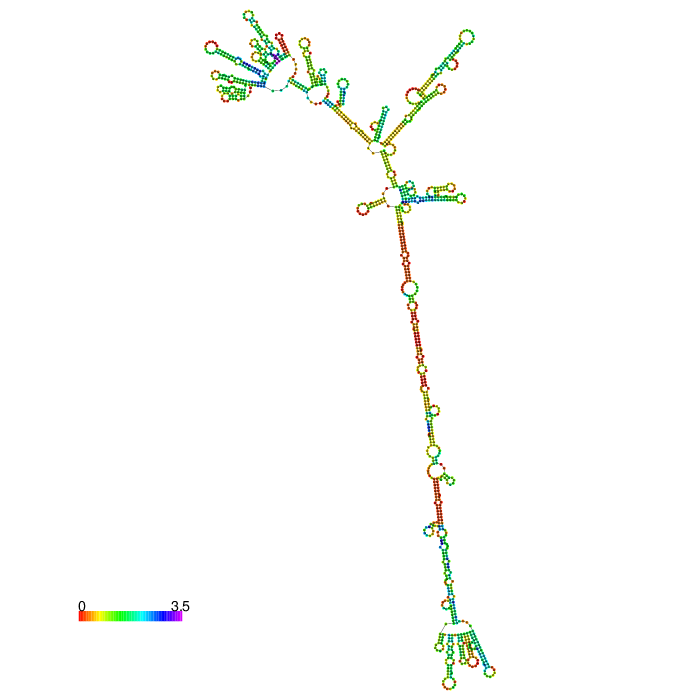

Supplement: Supplementary file 5 — Additional file 5: Fig. S2. Minimum free energy structure of mRNA encoding its position entropy. [file 42269_2023_1013_MOESM5_ESM.docx]

**Supplementary figure 3.** Centroid structure of mRNA encoding its position entropy.


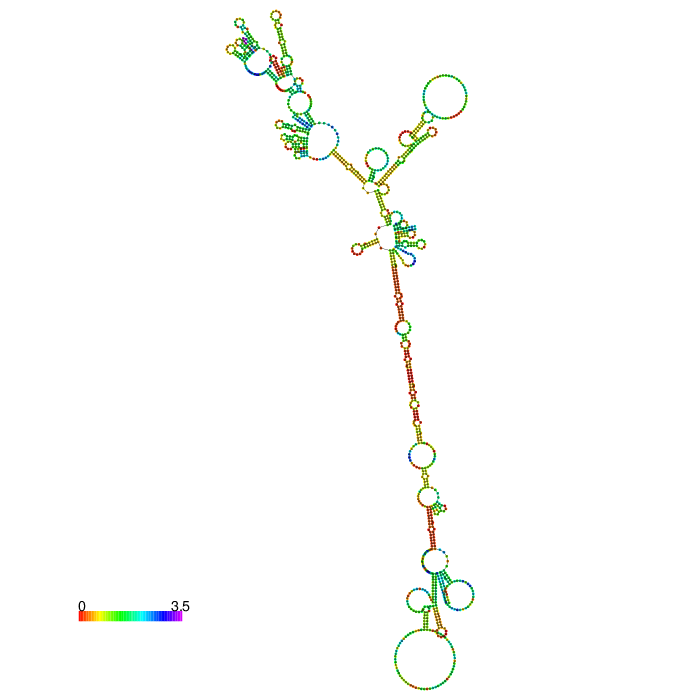

Supplement: Supplementary file 6 — Additional file 6: Fig. S3. Centroid structure of mRNA encoding its position entropy. [file 42269_2023_1013_MOESM6_ESM.docx]
