# Supplementary material for: Construction and validation of a multi-epitope in silico vaccine model for lymphatic filariasis by targeting Brugia malayi: a reverse vaccinology approach
Source: Bull Natl Res Cent. 2023 Mar 24;47(1):47. doi: 10.1186/s42269-023-01013-0 (PMC10037386; doi:10.1186/s42269-023-01013-0)

**Supplementary figure 4.** A mountain plot of minimum free energy structure, the thermodynamic ensemble of RNA structures, and the centroid structure along with a graphical illustration of the positional entropy at each position.


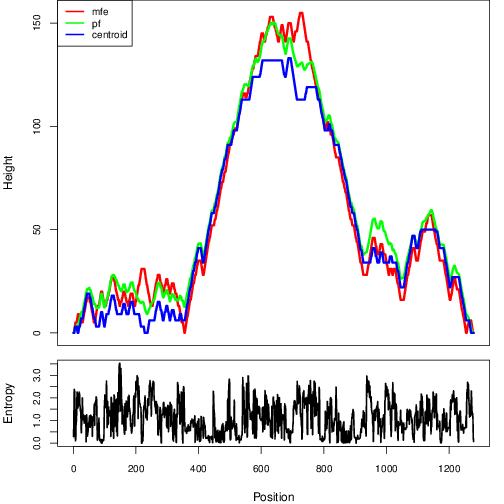

Supplement: Supplementary file 7 — Additional file 7: Fig. S4. A mountain plot of minimum free energy structure, the thermodynamic ensemble of RNA structures, and the centroid structure along with a graphical illustration of the positional entropy at each position. [file 42269_2023_1013_MOESM7_ESM.docx]
